# TeamMate: a longitudinal study of New Zealand working farm dogs. I. Methods, population characteristics and health on enrolment

**DOI:** 10.1186/s12917-020-2273-2

**Published:** 2020-02-17

**Authors:** Katja E. Isaksen, Lori Linney, Helen Williamson, Nick J. Cave, Ngaio J. Beausoleil, Elizabeth J. Norman, Naomi Cogger

**Affiliations:** 1 School of Veterinary Science, Massey University, Palmerston North, New Zealand; 2 Vetlife, 28 Sophia Street, Timaru, New Zealand; 3 College of Sciences, Massey University, Palmerston North, New Zealand

**Keywords:** Working dog, Working farm dog, Heading dog, Huntaway, Population survey, Health survey, Clinical examination, Prevalence, TeamMate

## Abstract

**Background:**

Working farm dogs are invaluable on New Zealand sheep and beef farms. To date no study describing farm dog population and health has included information about incidence of illness and injury, or risk factors affecting health and career duration. This paper describes the methodology and initial results from TeamMate, a longitudinal study that was designed to address this gap. We describe the study population, husbandry practices, and prevalence of clinical abnormalities on enrolment.

**Methods:**

Data about the farms, owners, husbandry practices and dogs were collected on farm at approximately 6-month intervals. All dogs over 18 months old and in full work were enrolled. Dogs were given physical examinations by veterinarians. On examination all abnormalities were noted, regardless of clinical significance.

**Results:**

Six hundred forty-one working farm dogs and 126 owners were enrolled from the South Island of New Zealand. Forty-nine percent of dogs were Heading dogs (314 of 641) and 48% Huntaways (308 of 641). Median age of dogs was 4 years (range 1.5–14) and median body condition score (BCS) was four on a 9-point scale (interquartile range (IQR) 3–5). Fifty-four percent of dogs were male (345 of 641), and 6% (41 of 641) were neutered.

Eighty-one percent of owners (102 of 126) fed dogs commercial biscuits and meat sourced on farm. Forty-four percent of dogs (279 of 641) had bedding in their kennel, 14% (55 of 393) had insulated kennels, 69% (442 of 641) had been vaccinated and 33% (213 of 641) were insured.

Clinical abnormalities were found in 74% of dogs (475 of 641). Common abnormalities involved the musculoskeletal system (43%, 273 of 641), skin (including scars and callouses; 42%, 272 of 641), and oral cavity (including worn and broken teeth; 35%, 227 of 641).

**Conclusions:**

Our results expand on those from previous surveys and indicate that musculoskeletal illness and injury, and skin trauma are the most commonly seen clinical abnormalities in working farm dogs. These results will provide a baseline for investigation of incidence and risk factors for illness, injury, retirement and death in New Zealand working farm dogs.

## Background

There are over 25,000 sheep and beef farms in New Zealand [1]. In 2016, meat and wool exports were worth NZ$6.7 billion, accounting for 14% of New Zealand’s total exports of goods. In 2016–17 New Zealand was the third largest wool exporter in the world, producing 9% of the world wool supply [2, 3]. Many of the sheep and beef farmers who supply these products rely heavily on dogs when mustering and moving stock between pastures, and it is often said that the rough New Zealand terrain could not be farmed without the help of dogs [4]. It has been estimated that there are approximately 200,000 working farm dogs in New Zealand, most of them belonging to one of two distinct types of dog [4, 5]. These dog types, called Heading dogs and Huntaways, are anecdotally known to be phenotypically distinct and having been bred to perform different types of stock work. However, no data is available to verify the population size, or the differences between the types of dogs.

Maintaining the health of working farm dogs is important for farmers who rely on their assistance, but little research has been conducted about the specific needs of these dogs. Today, husbandry practices are often based on traditional and anecdotal knowledge that is passed between dog owners and trainers or documented in training manuals [6–8]. Other sources are studies of health in pet dogs and other types of working dogs such as military and police dogs, assistance dogs for the disabled or racing dogs [9–13]. However, advice that is well founded and useful for pet dogs and other types of working dogs may not be applicable to highly athletic working farm dogs that live most of their lives outdoors. Advice on husbandry practices needs to be based on sound evidence that the recommended changes are likely to improve the health, welfare and career longevity of working farm dogs specifically. Currently, such evidence does not exist.

Previous surveys have described sections of the farm dog population [14–17]. Two of these studies reported farm dog health, with Sheard surveying owners about the health of their dogs in the previous year [15] and Cave et al. recording farm dog visits to 30 veterinary clinics during the course of 1 year [14]. Sheard [15] relied completely on owner reports, which may be unreliable, and did not collect clinical data from the dogs. Cave et al. [14] analysed records of farm dogs that were seen in veterinary clinics, but had no way to record health events that happened on farm and were not seen by a veterinarian. This will have resulted in an under-reporting of cases that were judged by the owner to not warrant a visit to a veterinary clinic and cases where the problem was resolved or the dog died before being seen by a veterinarian. When taken together the two studies provide valuable data about the health of farm dogs, but they have major limitations, mostly due to sampling bias. Additionally, the authors were unable to record how disease develops over time or investigate risk factors that may affect the likelihood of disease, retirement or death. A longitudinal study collecting clinical data about dogs at several points over a period of time would be able to investigate whether or not certain factors were present before the onset of disease, and whether their presence increased the risk of a dog developing disease. Knowledge about such risk factors would be very useful to farm dog owners and veterinarians making decisions about how to provide the best care for farm dogs.

This paper describes TeamMate, a longitudinal study of 641 working farm dogs on the South Island of New Zealand. TeamMate was designed to accomplish a number of objectives: (1) to gather population data on working farm dogs on the South Island; (2) to identify common husbandry practices; (3) to supplement current knowledge about common injuries and diseases occurring in farm dogs; (4) to gather data regarding the work farm dogs are required to carry out; and (5) to investigate how the above factors interact with, and contribute to, the long-term health and career longevity of working farm dogs. The aims of the present paper are to describe the design, implementation and the population involved in the TeamMate study. Specifically, we describe the farms and dog owners involved in the study. Then, we describe the population of working farm dogs and their feeding, shelter and vaccination status. Lastly, we report the prevalence of abnormalities found on each dog’s initial clinical exam on enrolment in the study.

## Results

**Fig. 1:**
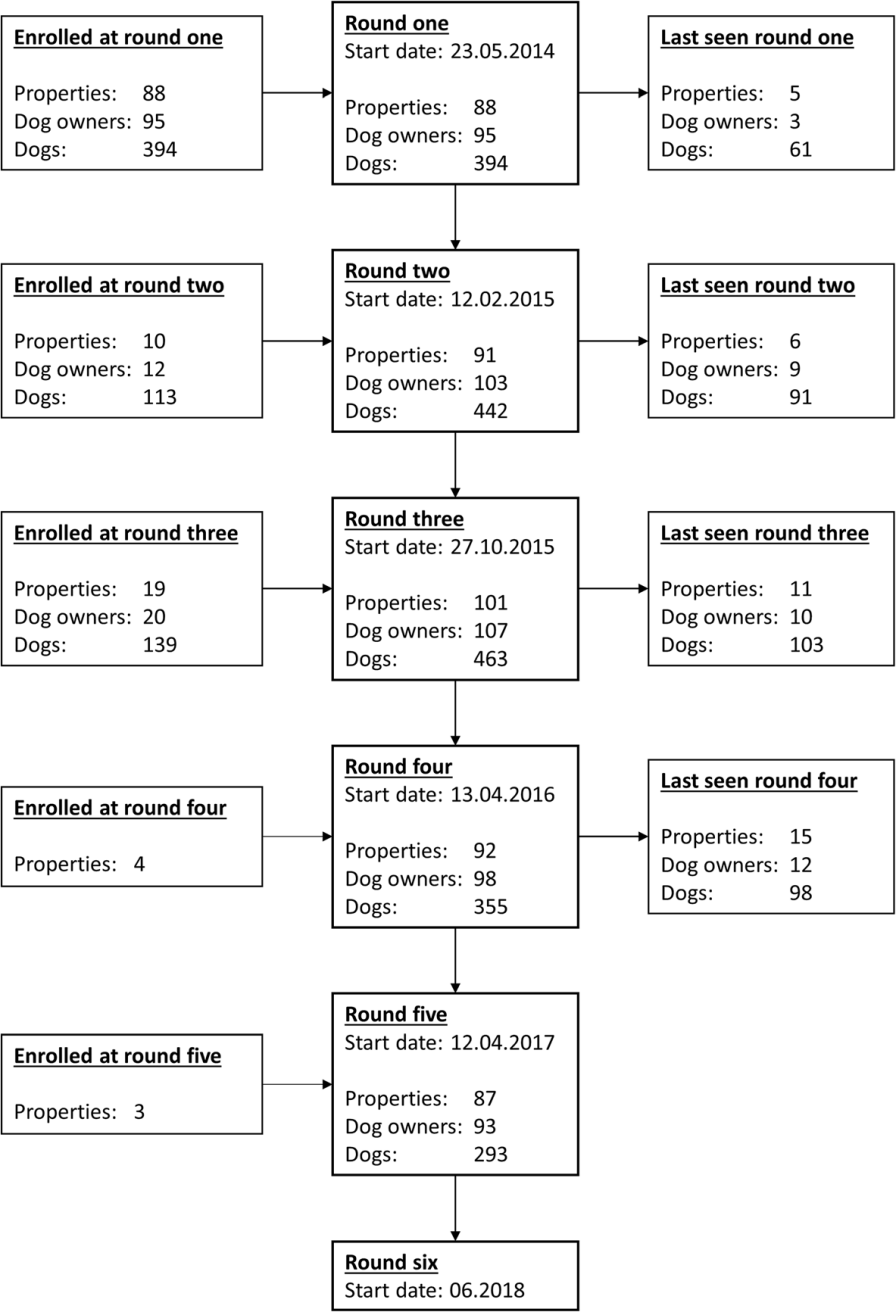
Flow chart showing the start dates of each data collection round as well as the number of farms, dog owners and dogs enrolled in TeamMate up to and including the fifth round of farm visits. Additionally, 14 properties, 16 dog owners and 68 dogs missed at least one round of data collection. Note that data for the sixth data collection round was not yet available at the time of writing

Data collection for TeamMate was carried out over a period spanning May 2014 to the second half of 2018 (Fig. 1). Enrolment of dog owners and dogs was completed in May 2016. In total 126 owners associated with 116 farms participated in the study and 641 working farm dogs were enrolled.

### Farming properties and dog owners

**Fig. 2:**
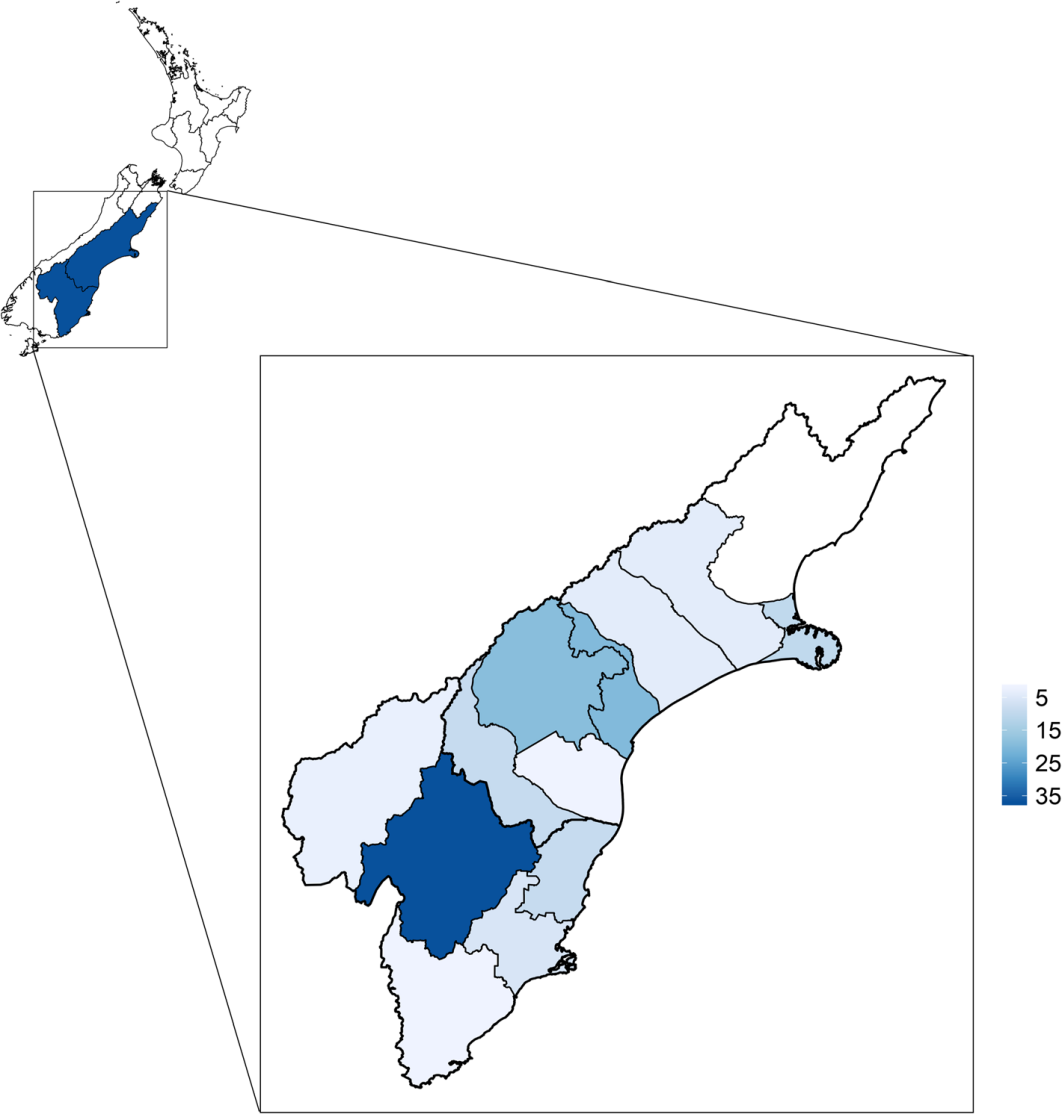
Map of New Zealand with the regions of Canterbury and Otago expanded. Shaded blue areas show the study area, with a darker shade indicating more farming properties. The study area is located between approximately − 46 and − 43 degrees longitude. The files used to generate this map were sourced from Stats NZ [18] and licensed by Stats NZ for re-use under the Creative Commons Attribution 4.0 International licence

All farms were located in Otago and Canterbury, on New Zealand’s South Island (Fig. 2).

Table 1 summarises the number of farms, dog owners and dogs by types of terrain and stock present on the property. The median property size was 1511 ha (IQR = 501–4500 ha). Stock types and numbers were reported for 115 farming properties. The total number of stock animals on farm ranged from 12 to 36,000 animals, with a median of 4320 stock animals per farming property (IQR = 2220–6350).

**Table 1:** Number and percentage of farms, owners and dogs stratified by terrain and type of stock present and combinations of stock present. Data were collected from 641 dogs, 126 dog owners and 116 farms that participated in TeamMate. Combinations of stock that were seen on fewer than 10 farms were combined and listed as ‘Other’. Percentages do not add up to 100% due to incomplete recording of data and because most properties, dog owners and dogs were associated with more than one type of stock

Property variables	Farms		Owners		Dogs	
	n	% (95% CI)	n	% (95% CI)	n	% (95% CI)
Terrain						
Both flat and steep	61	53 (43–62)	70	56 (47–64)	350	55 (51–58)
Flat only	34	29 (21–38)	34	27 (19–35)	159	25 (21–28)
Steep only	20	17 (10–24)	21	17 (10–23)	18	18 (15–21)
Type of stock present						
Sheep	111	96 (92–99)	121	96 (93–99)	16	96 (95–98)
Beef cattle	104	90 (84–95)	114	90 (85–96)	581	91 (88–93)
Dairy cattle (dry)	20	17 (10–24)	21	17 (10–23)	116	18 (15–21)
Deer	17	15 (8–21)	23	18 (12–25)	107	17 (14–20)
Other stock present	10	9 (4–14)	12	10 (4–15)	63	10 (8–12)
Combinations of stock						
Sheep and beef cattle	74	64 (55–73)	78	62 (53–70)	400	62 (59–66)
Sheep, beef cattle and dairy cattle (dry)	13	11 (5–17)	13	10 (5–16)	75	12 (9–14)
Sheep, beef cattle and deer	10	9 (4–14)	15	12 (6–18)	66	10 (8–13)
Other combinations of stock	19	16 (10–23)	20	16 (9–22)	100	16 (13–18)

**Fig. 3:**
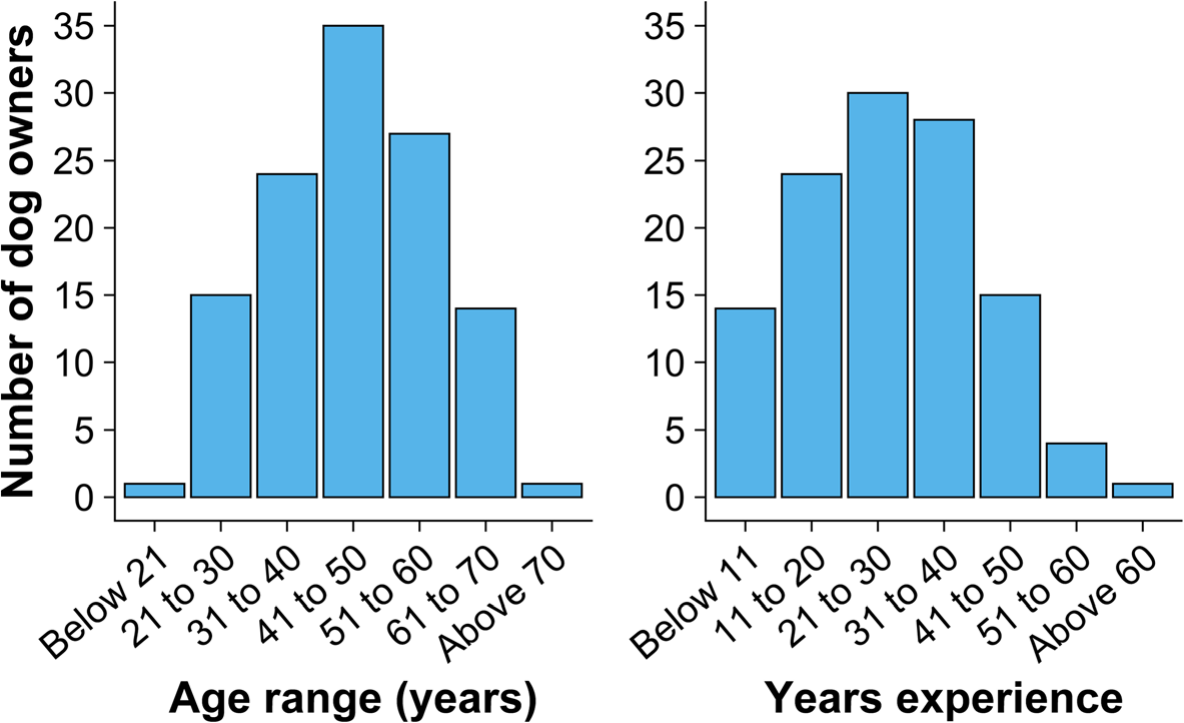
Left: Bar chart showing the number of dog owners stratified by age range (*n* = 117). Right: Bar chart showing the number of dog owners stratified by years of experience working with farm dogs (*n* = 116). Data were collected from working farm dog owners who participated in TeamMate

Eighty-four percent (106 of 126, 95% confidence interval (CI) = 78–91%) of working farm dog owners were male, 58% (66 of 113, 95% CI = 49–67%) were the farm owner, 19% (22 of 113, 95% CI = 12–27%) were the farm manager and 19% (21 of 113, 95% CI = 11–26%) were employees. Sixty-three percent (75 of 120, 95% CI = 54–71%) had participated in training relating to farm dogs. Figure 3 shows the owners’ age ranges and years of experience working with farm dogs. At the time of enrolment the median number of dogs per owner was four (range 1–9).

### Farm dogs

#### Population features

**Fig. 4:**
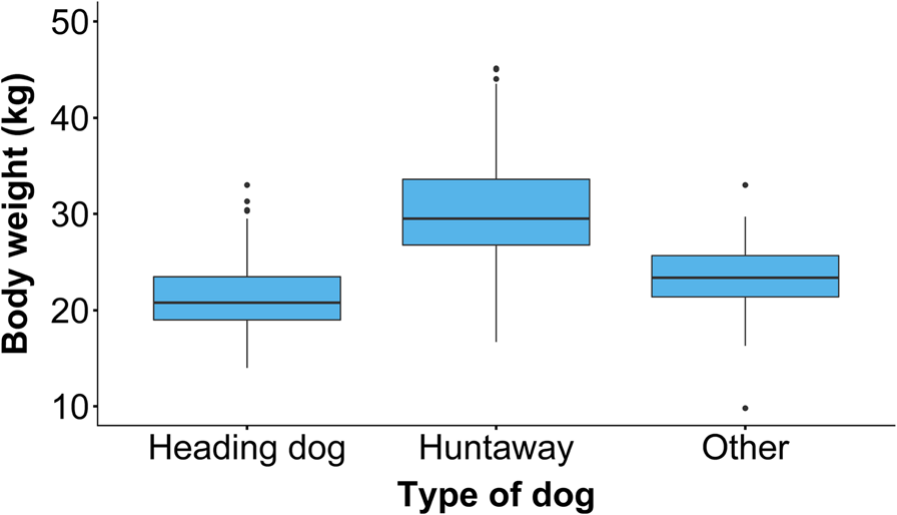
Boxplots showing the recorded body weights of 298 Heading dogs, 299 Huntaways and 19 dogs of other types. Data were collected from working farm dogs that were enrolled in TeamMate

Population features of all dogs enrolled in TeamMate are summarised in Table 2. The median age of enrolled dogs was 4 years (IQR = 2–6). Mean body weight across all enrolled dogs was 26 kg (*n* = 608, SD = 6), with some differences seen between types of dogs (Fig. 4). Median BCS was four out of nine (*n* = 634, IQR = 3–5), with a range of one to seven.

**Table 2:** Number and percentage of dogs stratified by type of dog, sex and neuter status, age and source of the dog. Data were collected from 641 working farm dogs enrolled in TeamMate

Population features	Dogs	% (95% CI)
Type of dog		
Heading dog	314	49(45–53)
Huntaway	308	48 (44–52)
Handy dog	13	2 (1–3)
Kelpie	3	0 (0–1)
Other mixed breed	2	0 (0–1)
Unknown	1	0 (0–0)
Sex and neutering		
Female entire	250	39 (35–43)
Female neutered	29	5(3–6)
Female neuter status unknown	17	3(1–4)
Male entire	305	48 (44–51)
Male neutered	12	2(1–3)
Male neuter status unknown	28	4(3–6)
Age range		
1.5 to 3 years	291	45 (42–49)
3.1 to 5 years	143	22 (19–26)
5.1 to 7 years	94	15 (12–17)
7.1 to 10 years	92	14 (12–17)
Above 10 years	21	3(2–5)
Source of dog		
Obtained from another breeder	466	73 (69–76)
Bred by current owner	148	23 (20–26)
On loan	1	0 (0–0)
Not recorded	26	4

More females than males were neutered (Table 2). In females, 15 dogs were reported to have been neutered due to medical issues such as vaginal prolapse, pyometra or problems with pregnancy or whelping, four to prevent unwanted pregnancies and two due to their temperament. It should be noted that though seven female dogs were reported to have been neutered due to ‘prolapse’ or ‘vaginal prolapse’, these cases are more likely to be mis-identified cases of vaginal hyperplasia. In fact, one case was reported as ‘Prolapse / vaginal hyperplasia’. Four males were reported to have been neutered due to unspecified behavioural issues, four to prevent fighting and unwanted mating, three due to prostate disease and one to both stop mating and correct an unspecified body weight issue. Six females and one male had no recorded reason for being neutered.

The main modes of work New Zealand working dogs are trained to carry out are outlined in Table 3, and the distribution of working roles between the Heading dog and Huntaway types of dog is seen in Table 4. Note that ‘Heading dog’ and ‘Huntaway’ refers to the type of dog, while ‘Head’ and ‘Hunt’ refers to specific tasks carried out by working farm dogs. While the naming of the dog types is related to the work these dogs normally do, there is an amount of overlap in the tasks dogs in this dataset have been trained to carry out.

**Table 3:** An overview of the modes of work commonly done by New Zealand working farm dogs. Dogs can be trained to carry out one or several modes of work

Mode of work	Description
Head	The dog circles around to the head of the herd and uses its positioning to gather, stop and redirect animals. This type of work is typically, but not exclusively, carried out by Heading dogs.
Hunt	The dog uses its bark and position to apply pressure to the herd from behind in order to move the animals forward. This type of work is typically, but not exclusively, carried out by Huntaways.
Yard work	Any work done in stockyards and runs.
Catch	Separating one or several specific animals from the herd.

**Table 4:** Number and percentage (with 95% CI) of Heading dogs (*n* = 314) and Huntaways (*n* = 308) stratified by the ways in which they were trained to move stock. Data were collected from 641 working farm dogs enrolled in TeamMate. Percentages do not add up to 100% as many dogs were trained to carry out more than one mode of work

Mode of work	Heading dogs		Huntaways	
	n	% (95% CI)	n	% (95% CI)
Head	291	93 (90–96)	81	26 (21–31)
Hunt	17	5 (3–8)	284	92 (89–95)
Yard work	52	17 (12–21)	253	82 (78–86)
Catch	132	42 (37–48)	44	14 (10–18)
Not reported	17	5	16	5

**Fig. 5:**
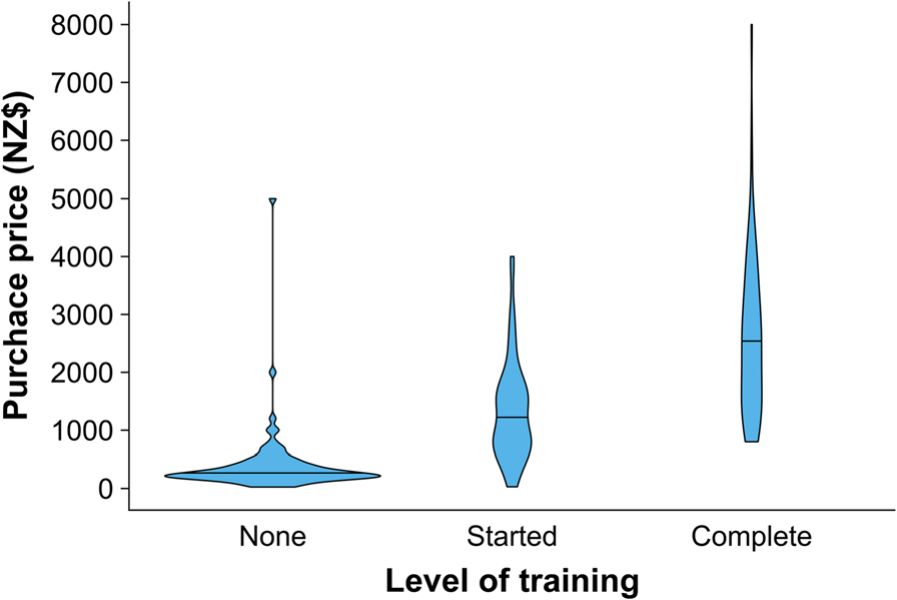
Violin plot, with the mean indicated, showing the purchase price of 200 working farm dogs stratified by level of training. Dogs that were acquired at no cost or had unknown purchase price were not included. Data were collected from working farm dogs that were enrolled in TeamMate

Table 2 shows the origins of all dogs in the study. Of the 466 dogs that had been acquired from another person, money was exchanged for 216 or 46% (95% CI = 42–51%). One hundred eighty-two of the remaining dogs were given at no cost and 51 dogs were traded. Trades involved alcohol (typically cases of beer), exchanging for another dog, or various other items. The median age at acquisition was 12 weeks (*n* = 466, IQR = 8–104). Fifty-four percent (*n* = 250 of 466, 95% CI = 49–58%) of the dogs had received no training prior to arriving with their current owner, 22% (*n* = 102, 95% CI = 18–26%) had been partly trained and 19% (*n* = 90, 95% CI = 16–23%) were fully trained. Twenty-four dogs had no record of their level of training on arrival. Across all purchased dogs in which money was exchanged the median price was NZ$800 (*n* = 216, range = NZ$26 to NZ$8000). Figure 5 illustrates the range of purchase prices stratified by level of training at the time of purchase.

#### Husbandry practices and feeding

Table 5 summarises a range of variables related to husbandry and housing of enrolled dogs. Of 34 dogs that did not have bedding in their kennel five were noted to have rejected the bedding provided to them and 13 to have bedding in winter. Six of the 333 dogs that were reported to not wear a coat were reported to have rejected it.

**Table 5:** Number and percentage of working farm dogs (*n* = 641) stratified by health management, registration status and housing. Data were collected from 641 working farm dogs enrolled in TeamMate. Details about kennel construction were obtained in relation to 393 dogs that were enrolled during the first round of farm visits. Percentages do not add up to 100% because of incomplete recording of data

Variables	Dogs	% (95% CI)
Vaccination status		
Only vaccinated as pup	290	45 (41–49)
Never vaccinated	77	12 (9–15)
Interval other than yearly	61	10 (7–12)
Yearly	58	9 (7–11)
Sporadically	33	5 (3–7)
Owner unsure of vaccination status	65	10 (8–12)
Dog insured		
Yes	213	33 (30–37)
No	373	58 (54–62)
Council registration		
Yes	418	65 (62–69)
No	128	20 (17–23)
Wears a coat		
Yes	154	24 (21–27)
No	333	52 (48–56)
Bedding in kennel provided		
Yes	279	44 (40–47)
No	311	49 (45–52)
Kennel construction		
Source of kennels		
Commercial	266	68 (63–72)
Home–made	120	31 (26–35)
Kennel type		
Motel with individual run	282	72 (67–76)
Kennel with chain	104	26 (22–31)
Other	3	1
Kennel elevated from ground		
Yes	362	92 (89–85)
No	19	5 (3–7)
Kennel insulated		
Yes	55	14 (11–17)
No	320	81 (78–85)

Table 6 shows the types of food dogs had been fed in the 6 months prior to enrolment in the study, and Table 7 shows what combinations of foods dogs were fed at their most recent meal prior to enrolment. ‘Meat sourced on farm’ refers to livestock, and occasionally game animals, that have been killed and butchered on farm.

**Table 6:** The numbers and percentages of dog owners stratified by the types of foods they reported to have given to their working farm dogs during a 6–month period. Data were collected from 126 working farm dog owners participating in TeamMate. Note that percentages do not add up to 100% because many owners fed more than one type of food

Food fed to dogs	Owners	% (95% CI)
Meat	107	85 (79–91)
Source		
Sourced on farm	105	83 (77–90)
Purchased	16	13 (7–19)
Treatment		
Frozen	100	79 (72–86)
Fresh	27	21 (14–28)
Offal	28	22 (15–29)
Cooked	25	20 (13–27)
Fresh	1	1(0–2)
Commercial dog food	113	90 (84–95)
Dry dog food	111	88 (82–94)
Wet dog food	54	43 (34–51)
Other commercial food	27	21 (14–29)
Not recorded	8	6 (2–11)

**Table 7:** The number and percentage of working farm dogs stratified by the types of foods comprising their most recent meal at the time of their enrolment to the study. Data were collected from 641 working farm dogs enrolled in TeamMate. Combinations of foods that were fed to fewer than 10 dogs are combined and listed as ‘Other combinations’

Most recent meal	Dogs	% (95% CI)
Meat only	242	38 (34–42)
Dry commercial food only	207	32 (29–36)
Meat and dry dog food	85	13 (11–16)
Dry and wet dog foods	25	4 (2–5)
Wet dog food only	14	2 (1–3)
Meat, dry and wet dog foods	13	2 (1–3)
Dry and other commercial foods	10	2 (1–3)
Other combinations	30	5 (3–6)

#### Clinical examination

The prevalence of abnormal findings in each of the main categories, and the prevalence of abnormalities in Heading dogs and Huntaways can be seen in Table 8. For those dogs in which at least one abnormality was recorded, the median number of abnormalities per dog was three (IQR = 1–4). Note that recorded abnormalities in this study include anything that deviates from the ideal, including signs of previously healed injuries and normal wear that do not necessarily represent reduced health or welfare at the time of examination. As no clear differences were seen between Heading dogs and Huntaways in the prevalence of the major types of abnormalities, the remaining results are presented for the entire population without stratification by type of dog.

**Table 8:** The number and percentage of dogs that were recorded to have at least one abnormal clinical finding, stratified by body system. Numbers and percentages are shown for the entire population (n = 641) along with numbers and percentages of the two main types of dog: Heading dogs (n = 314) and Huntaways (n = 308). Data were collected from 641 working farm dogs that were enrolled in TeamMate. Percentages do not add up to 100% as many dogs were recorded to have more than one type of clinical abnormality

Type of abnormality	All dogs		Heading dogs		Huntaways	
	n	% (95% CI)	n	% (95% CI)	n	% (95% CI)
Musculoskeletal	272	42 (39–46)	121	39 (33–44)	143	46 (41–52)
Skin	272	42 (39–46)	129	41 (36–47)	138	45 (39–50)
Oropharyngeal	227	35 (32–39)	122	39 (33–44)	96	31 (26–36)
Ocular	66	10 (8–13)	35	11 (8–15)	30	10 (6–13)
Reproductive	45	7 (5–9)	15	5 (2–7)	27	9 (6–12)
Lymph nodes	9	1 (0–2)	2	1 (0–2)	6	2 (0–3)
Heart	6	1 (0–2)	5	2 (0–3)	1	0 (0–1)
Hernia	3	0 (0–1)	2	1 (0–2)	1	0 (0–1)
Respiratory	2	0 (0–1)	1	0 (0–1)	1	0 (0–1)
Gastrointestinal	1	0 (0–0)	1	0 (0–1)	1	0 (0–1)
Urinary	1	0 (0–0)	1	0 (0–1)	0	0
Other	3	1 (0–1)	3	1 (0–2)	0	0
Any abnormality	476	74 (71–78)	230	73 (68–78)	235	76 (72–81)

Twenty-nine percent of dogs (183 of 641, 95% CI = 25–32%) had at least one musculoskeletal abnormality in the hind limbs, 20% of dogs (130 of 641, 95% CI = 17–23%) had an abnormality in the front limbs and 7% of dogs (44 of 641, 95% CI = 5–9%) had an abnormality in the spine or tail. Lameness on trot was observed in 12% of all dogs (83 of 641, 95% CI = 10–16%) or 26% of dogs with a musculoskeletal abnormality (78 of 272, 95% CI = 21–31%). Table 9 and Table 10 show the prevalence of a range of musculoskeletal abnormalities in the forelimbs and hind quarters (including the tail), and the number of those dogs that were also lame when trotted up. Twenty-three dogs (*n* = 641, 4, 95% CI = 2–5%) had abnormalities relating to the ribs and spine (excluding the tail) that could not be categorised as belonging to the front or hind part of the body. They are therefore not represented in the tables. Twenty-one dogs (n = 641, 3, 95% CI = 2–5%) showed signs of pain on manipulation of the spine. Ten of these 21 dogs were also lame on trot-up (48, 95% CI = 26–69%). One dog was recorded to have a swelling at the sacroiliac joint and to also be lame in the hind quarters. Additionally, one dog had an abnormal curvature of the lumbar spine, and one dog was recorded to have a protruding 13th rib on the left side. These two dogs were not observed to be lame.

**Table 9:** The number and percentage of working farm dogs with reported musculoskeletal abnormalities in the front quarters, and the number and percentage of dogs with musculoskeletal abnormalities that were also lame in the front quarters. Data were collected from 641 working farm dogs that were enrolled in TeamMate. Types of clinical abnormalities that were recorded in fewer than 10 dogs are combined and listed as ‘Other’

Anatomical location and type of abnormality	Number of dogs		Lame front leg(s)	
	n	% (95% CI)	n	% (95% CI)
Shoulder				
Reduced range of motion	15	2 (1–4)	2	13 (0–31)
Other	9	1 (0–2)	2	22 (0–49)
All dogs with shoulder abnormalities	23	4 (2–5)	4	17 (2–33)
Elbow				
Reduced range of motion	11	2 (1–3)	2	18 (0–41)
Other	25	4 (2–5)	5	20 (4–36)
All dogs with elbow abnormalities	31	5 (3–6)	6	19 (5–33)
Carpals				
Crepitus	10	2 (1–3)	4	40 (10–70)
Reduced range of motion	52	8 (6–10)	10	19 (9–30)
Hard swelling	11	2 (1–3)	2	18 (0–41)
Other	16	2 (1–4)	5	31 (9–54)
All dogs with carpal abnormalities	69	11 (8–13)	15	22 (12–31)
Metacarpals				
Other	4	1 (0–1)	0	0
All dogs with metacarpal abnormalities	4	1 (0–1)	0	0
Front digits				
Hard swelling	14	2 (1–3)	1	7 (0–21)
Other	16	2 (1–4)	4	25 (4–46)
All dogs with front digit abnormalities	26	4 (3–6)	4	15 (2–29)

**Table 10:** The number and percentage of working farm dogs with reported musculoskeletal abnormalities in the hind quarters (including tail), and the number and percentage of dogs with a musculoskeletal abnormality that were also lame in the hind quarters. Data were collected from 641 working farm dogs that were enrolled in TeamMate. Types of clinical abnormalities that were recorded in fewer than 10 dogs are combined and listed as ‘Other’

Anatomical location and type of abnormalities	Number of dogs		Lame hind leg(s)	
	n	% (95% CI)	n	% (95% CI)
Hip				
Reduced range of motion	59	9 (7–11)	12	20 (10–31)
Painful	43	7 (5–9)	15	35 (21–49)
Other	8	1 (0–2)	4	50 (15–85)
All dogs with hip abnormalities	88	14 (11–16)	21	24 (15–33)
Stifle				
Crepitus	19	3 (2–4)	5	26 (7–46)
Reduced range of motion	18	3 (2–4)	5	28 (7–48)
Hard swelling	32	5 (3–7)	6	19 (5–32)
Other	13	2 (1–3)	4	31 (6–56)
All dogs with stifle abnormalities	62	10 (7–12)	16	26 (15–37)
Tarsals				
Reduced range of motion	27	4 (3–6)	8	30 (12–47)
Hard swelling	12	2 (1–3)	4	33 (7–60)
Other	10	2 (1–3)	1	10 (0–29)
All dogs with tarsal abnormalities	43	7 (5–9)	9	21 (9–33)
Tail				
Reduced range of motion	12	2 (1–3)	1	8 (0–24)
Other	12	2 (1–3)	2	17 (0–38)
All dogs with tail abnormalities	22	3 (2–5)	3	14 (0–28)
Hind digits				
Other	19	3 (2–4)	4	21 (3–39)
All dogs with hind digit abnormalities	19	3 (2–4)	4	21 (3–39)
Metatarsals				
Other	6	1 (0–2)	1	17 (0–46)
All dogs with metatarsal abnormalities	6	1 (0–2)	1	17 (0–46)

Table 11 shows the prevalence of the different types of skin, eye and reproductive system abnormalities. Fifty-eight dogs (n = 641, 9%, 95% CI = 7–11%) had a callous and/or a healed scar with no other skin abnormality present. Ninety-three percent of dogs with skin callouses had them on the legs (93 of 100, 95% CI = 88–98%). Of dogs with a healed scar, an open or healing wound or both, 65% (100 of 153, 95% CI = 58–73%) had them on the face or ear, 35% (53 of 153, 95% CI = 27–42%) on the legs, 11% (17 of 153, 95% CI = 6–16%) on the torso, 8% (12 of 153, 95% CI = 4–12%) on the foot (including nails) and one on the tail. Types of skin abnormalities categorised as ‘Other’ included six dogs with missing nails, eight with poor coat condition, three that were missing part of an ear, two with pruritus, and one dog that had abnormal wear of the nails on one foot.

**Table 11:** The number and percentage of working farm dogs with reported abnormal findings associated with the skin, eyes and reproductive systems. Data were collected from 641 working farm dogs that were enrolled in TeamMate. Types of clinical abnormalities that were recorded in fewer than 10 dogs are combined and listed as ‘Other’. Note that dogs could be recorded to have more than one clinical abnormality

Type of abnormal finding	Dogs	% (95% CI)
Skin		
Callous	100	16 (13–18)
Scar	98	15 (13–18)
Laceration	68	11 (8–13)
Inflammation	31	5 (3–6)
Mass	30	5 (3–6)
Alopecia	28	4 (3–6)
Infection	12	2 (1–3)
Other	21	3 (2–5)
Eyes		
Opacity	37	6 (4–8)
Scarring	10	2 (1–3)
Other	25	4 (2–5)
Reproductive system		
Mammary tumour	21	3 (2–5)
Other	24	4 (2–5)

Table 12 shows the prevalence and placement of recorded clinical abnormalities relating to the teeth. Abnormalities classed as ‘Other’ included eight dogs with periodontitis or tooth abscesses, three dogs were observed to have a focal enamel defect and one had several retained juvenile incisors. Additionally, three dogs had gingivitis and two dogs had soft tissue injuries in the mouth.

**Table 12:** The number and percentage of working farm dogs that were recorded to have clinical abnormalities related to the teeth. Types of abnormalities are shown stratified by location in the mouth as well as combined. Data were collected from 641 working farm dogs that were enrolled in TeamMate. Types of clinical abnormalities that were recorded in fewer than 10 dogs are combined and listed as ‘Other’. Note that dogs could be recorded to have more than one tooth abnormality

Type of abnormal finding	Front teeth	Back teeth	General	All locations	
	n	n	n	n	% (95% CI)
Tooth fracture(s)	84	13	7	104	16 (13–19)
Tooth wear	55	8	17	80	12 (10–15)
Tooth / teeth missing	36	2	4	42	7 (5–8)
Tartar	2	3	21	26	4 (3–6)
Malocclusion	1	1	17	18	3 (2–4)
Tooth discolouration	9	1	1	11	2 (1–3)
Other	8	4	0	12	2 (1–3)

Ocular abnormalities categorised in Table 11 as ‘Other’ included four dogs with conjunctivitis, two with evidence of uveitis, and one with signs of both conjunctivitis and uveitis. Four dogs had tumours related to the meibomian gland, seven were blind or had reduced vision, two had brown discolouration of the iris, two had corneal ulcers, two had one missing eye, two had conjunctival discharge, and one dog had a unilateral deformity of the third eyelid.

Nineteen females and one male dog were recorded to have mammary tumours. In females, reproductive system abnormalities classed in Table 11 as ‘Other’ included nine females with mammary hyperplasia, two dogs with an extra nipple, one dog with vaginal discharge 8 weeks post whelping, and one dog was recorded to have vaginal prolapse. As mentioned above, the case reported as vaginal prolapse is likely to be a mis-characterised case of vaginal hyperplasia. In males, six dogs were cryptorchid, three had testes of unequal size, one had an enlarged prostate, one had scar tissue on the penis and one dog was described as having ‘small, soft testicles’.

Four dogs had one swollen popliteal lymph node, three had one or two swollen prescapular lymph nodes, one had one swollen mandibular lymph node and one dog had one swollen inguinal lymph node. Four dogs had an unclassified heart arrhythmia and two had a heart murmur. One dog had been diagnosed with a diaphragmatic hernia following an accident and one had a slight unilateral wheeze on auscultation. Three dogs had umbilical hernias, one dog had an anal gland abscess and one was reported to have haematuria.

## Discussion

The aim of the TeamMate project is to investigate health, career duration and loss of dogs over time. This initial paper describes the 641 working farm dogs that were enrolled in the study, their owners’ feeding and husbandry practices, their work, population features, and prevalence of abnormal findings on clinical examination.

Dogs were almost equally divided between males and females, and almost all dogs belonged to the Heading dog and Huntaway types, with only 19 of the 641 enrolled dogs classified as another type. We saw a clear division in the types of work done by Heading dogs and Huntaways, with Heading dogs mostly used to head and Huntaways mostly used to hunt. The differences seen in working roles between dogs described as Heading dogs and Huntaways in this study were expected, as these dogs are generally used for different types of stock work [4, 6, 8, 19, 20]. However, there was a degree of overlap, suggesting that the division of work is not the only criteria used to define a dog as a Heading dog or Huntaway. Heading dogs and Huntaways do not have defined phenotypes, pedigrees or genetics in the way that conventional dog breeds do. Consequently, we made the decision to avoid using the word ‘breed’ when referring to them. Although Heading dogs and Huntaways can be recognised based on appearance, their phenotypes are said to vary widely [4, 19, 20]. Generally, Heading dogs resemble short-haired Border Collies, from which they are thought to descend. They are mainly trained to ‘head’ and often to ‘catch’ which puts them at closer proximity to stock than Huntaways when moving stock in open areas (see Table 4 for definitions of work). Huntaways tend to be heavier than Heading dogs (Fig. 4), and have different colouring and more variability in coat length. They are trained to use their loud barks to drive or ‘hunt’ stock, and they are often used in yard work, which is a more confined environment. Huntaways are used for fine manoeuvring of stock less often than Heading dogs. Instead, they are used to apply pressure from behind and keep the herd moving while Heading dogs direct where they should go. When used as teams, Heading dogs and Huntaways can move large herds very effectively across long distances. However, the differences in the ways they work may put them at risk of developing different types of injuries. While no major differences between types of dogs were seen in the prevalence of clinical abnormalities, this will be re-examined when analysing data on the incidence of new abnormalities on follow-up and the rate of dogs that were lost from the workforce during the study period.

Seventy-five percent of dogs had at least one abnormal finding on clinical examination. Musculoskeletal system, skin and teeth abnormalities were by far the most common, and were recorded in a higher proportion of dogs than in the surveys by Sheard [15] and Cave et al. [14]. This is to be expected, as TeamMate was deliberately designed to capture all abnormalities in dogs and not just ones that were clinically significant at the time the data were collected. The earlier surveys recorded instances of illness or injury that were serious enough that owners thought to report them at a later date, or took the dogs to be seen by a veterinarian. Unlike these surveys, in TeamMate the term ‘abnormality’ encompasses any change to a dog, including healed scars, callouses and minor tooth wear that are unlikely to be considered a problem by the owner, or to directly impact on dogs’ health and welfare. However, these abnormalities illustrate the most common types of problems working farm dogs are likely to acquire, and they may be contributing factors to subsequent disease, retirement or death.

Several veterinarians participated in data collection, creating a possibility that different individuals assessed and described similar types of abnormalities in in different ways. However, in order to minimise bias in the data, veterinarians were asked to describe physical signs rather than to give overall diagnoses. While differences in data collected by different veterinarians are impossible to rule out, we have worked to minimised the risk of bias through our data collection, coding and data entry procedures. Additionally, a random sampling procedure may have resulted in a sample that was more representative of the farm dog population as a whole. However, in order to avoid a low response rate and to enable data collection to be carried out in a timely manner, a convenience sample of existing Vetlife clients was chosen.

Thirty percent of dogs in TeamMate were given a body condition score of three or below which places them in the ‘under ideal’ range according to the World Small Animal Veterinary Association [21]. This is in general agreement with Sheard’s data that dog owners considered one in five of their dogs to be underweight [15]. However, in Sheard’s study no data on body weight or body condition scores were collected that could have confirmed or negated owners’ assessments. O’Connell et al. [5] reports similarly low BCS in their sample population, but did not find a correlation between BCS and the presence of parasites in faecal samples, or dogs’ sex, age or housing. It should be noted that body condition scoring for dogs was developed with the aim of estimating body fat in overweight dogs [22, 23] and is poorly validated for athletic, lean dogs. In such dogs, loss of muscle mass may be a more relevant cause for concern. The ratio of lean body mass to skeletal size may be a better way to assess condition in lean dogs than BCS [24], although it remains to be seen which method has the greatest utility. Nonetheless, it has not been established what the ideal BCS or lean mass for a working farm dog is, or whether there are proportions of body fat or lean mass associated with an increased risk of disease or injury. An aim of the wider TeamMate project is to use the longitudinal data to investigate whether BCS and lean mass in farm dogs is related to injury, disease, or loss from work.

Similarly to dogs surveyed by Singh et al. and O’Connell et al. [5, 17] most dogs in this study were fed a combination of meat sourced on the farm and commercial dog food, and only one dog owner reported to having feed their dogs only meat in the previous 6 months. However, we were not able to record the amounts of food given, the quality of the food or the ratios of meat to commercial food. As such it is impossible to comment on whether the food given was adequate to their needs. However, un-supplemented meat is deficient in several minerals and vitamins [25], and if it is fed as the main proportion of the diet rather than as a supplement to a complete and balanced diet, it may result in malnutrition. To determine whether current feeding practices are associated with disease, injury or shortened lifespans, more detailed information is needed about the dogs, their energy expenditure and the exact size and composition of their meals. Most dog owners reported that the meat fed to dogs had been frozen, and that offal had been cooked. Working farm dogs are at risk of infection from a range of parasites that could be spread through untreated meat [5]. In addition to regular anthelmintic treatment, freezing or cooking meat and offal that is to be fed to dogs is recommended to reduce the spread of these parasites, especially those that might be spread to livestock [5, 26].

Over 80% of dogs in this study were housed in un-insulated kennels, less than half had bedding in their kennel and at least half of all dogs did not wear a coat for warmth at the time of enrolment. A dog’s energy expenditure can be affected by the quality of its housing, as ambient temperatures have an impact on dogs’ energy requirements, both if they are too hot and too cold [27]. Dogs that are housed in warm kennels use less energy on thermoregulation, and consequently have lower energy requirements. The recommended range of ambient temperature in order to maintain health and welfare in laboratory dogs is 20 – 26 °C [28]. In comparison, temperatures on the South Island can drop to well below 0 °C in the winter months [29]. Additionally, it has been shown that low temperatures are associated with increased levels of stress hormones, while dogs housed in actively heated kennels tend to rest more [30]. Though there is a great deal of variety in their phenotypes, most working farm dogs in New Zealand have relatively short, smooth coats that are likely to offer limited protection from cold temperatures. In this respect, comparing them to laboratory dogs such as Beagles is not unreasonable. Due to their athleticism and high activity levels, farm dogs are also likely to have less insulating subcutaneous fat than most laboratory dogs. In addition to helping with thermoregulation, providing appropriate bedding can help with preventing pressure sores on dogs’ elbows and hocks [28]. Three out of every 20 dogs in this study were reported to have callouses that were probably caused by lying on hard surfaces. It should be noted that in this study some of the questions relating to housing had relatively low response rates, and that some dogs were noted to have rejected the coats and bedding provided to them. Nonetheless, improving the housing for working farm dogs could have a positive effect on their health, welfare and career longevity.

Only 6 % of farm dogs were reported to be neutered. Farm dog owners may have a desire to be able to breed from dogs that prove to be good workers, causing them to only neuter dogs if they have a specific reason to do so. The rate of neutering was twice as high in females than in males, with most females having been neutered due to medical issues. In comparison, most male dogs had been neutered to stop unwanted mating and behavioural issues such as fighting. Some dog owners noted that neutering a male was done due to having one male in an otherwise all-female team, which is likely to make it more difficult than usual to isolate females in heat. Cave et al. [14] found that 9 % of clinic presentations of farm dogs involved a reproductive issue. The majority of these were mismatings, with mammary neoplasia being the second most common. An increased rate of neutering would decrease the rate of mismatings and might also reduce injuries caused by males fighting. However, it is uncertain whether neutering is beneficial for dogs’ overall health beyond removing risk directly related to the testes, ovaries and uterus. Most of the reproductive system abnormalities recorded in this study were mammary tumours or mammary hyperplasia. In the past it was believed that neutering reduces the risk of mammary tumours in female dogs, but the evidence supporting this claim is of variable quality [31, 32].

In the TeamMate population, only 24% of dogs were vaccinated as adults, from yearly to sporadically, although another 45% were known to have been vaccinated as a puppy. In comparison, a study of 196 working farm dogs on the North Island of New Zealand reported that 53% of dogs were vaccinated annually or every 2 years [5]. The majority of dogs in O’Connell et al.’s study were recruited from a veterinary practice in the Waikato region, with the remainder being recruited at a North Island sheepdog trial event [5]. Possibly, dog owners in Canterbury and Otago tend to live further from veterinary clinics than those in the Waikato region, making it more difficult for them to get their dogs vaccinated regularly. Additionally, dogs are barred from competing in trial events if they are ill with an infectious disease [33]. This may act as an incentive for owners to vaccinate their dogs.

For TeamMate we did not record the nature of the vaccines administered, though it is assumed the majority of vaccinations cover the core viral pathogens (distemper, adenovirus-2, parvovirus, ± parainfluenza). The duration of immunity elicited by the core vaccines is likely to extend beyond 3 years, and is probably life-long in many animals [34, 35]. Thus, it is very likely that a large proportion of dogs are sufficiently immunised against the core viral pathogens. Additionally, as farm dogs in New Zealand rarely move off the farm property, their risk of infection is much lower than in pet dogs. This is reflected in the low prevalence of suspected parvoviral enteritis in the study by Cave et al. [14]. Other vaccines that may be given to farm dogs include those protecting against leptospirosis and *Bordetella bronchiseptica*. Leptospirosis is common in New Zealand livestock, and seropositivity is relatively common in unvaccinated working farm dogs [36]. In addition, outbreaks of acute tracheobronchitis in working farm dogs have been seen by the authors, notably following trial meetings. Nonetheless, the significance of vaccination status to the health and career longevity of working farm dogs is not known. Depending on the results of future studies, more focus may need to be placed on ensuring appropriate vaccination coverage in working farm dogs.

Nearly 35% of farm dogs in the current study were covered by an insurance policy. This is higher than the 20% insurance coverage reported in Golden retrievers enrolled in a longitudinal study in the United States [37]. Additionally, in Australia it has been estimated that about 7% of dog owners have pet insurance [38], however it has been suggested that this might be an underestimate [39]. Due to the inherent differences between these populations, the validity of these comparisons could be disputed. However, little data is available on insurance rates in dogs, and no such data exists on pet dogs in New Zealand. The comparatively high rate of insurance in farm dogs might be explained by the fact that in New Zealand many insurance policies that cover assets related to farming also include the option to cover working farm dogs [40–42].

Over two thirds of participating farm dog owners were aged 30 to 59 years and three quarters reported having between 20 and 40 years of experience working with dogs, suggesting that those who work with farm dogs as adults often start learning at a very young age. A large majority of dog owners in our dataset report being the farm’s owner or manager, and very few recorded farms had more than one dog owner associated with it. Many farm managers employ farm hands and shepherds to help with the running of the farm. These shepherds usually own and work with their own team of dogs. As such, there is a possibility that there were dogs and owners working on participating farms that were not enrolled in TeamMate.

One aim of TeamMate was to try to gain a better understanding of the size of the farm dog population in New Zealand. Numbers are available on how many farming operations are present in New Zealand, but not on how many people on those farms own and work with dogs. However, due to the uncertainty surrounding the number of dogs and owners working on enrolled farms, we reported the median number of dogs per participating dog owner, not per farm. Previous studies have been unclear in whether they reported the number of dogs belonging to each owner surveyed or the number of dogs working on each farm. Most gathered data from a single owner on each property and reported the number of dogs per farm [15–17]. None mentioned whether or not other owners have worked with their dogs on the same farms. Jerram and Sheard analysed data from the same set of dogs, although Sheard included one less farm and 79 fewer dogs [15, 16]. They enrolled dogs from 6 months of age and included all dogs that had been working on the farm in the 12 months preceding the survey. Jerram reported a median of seven dogs and Sheard a mean of nine dogs per farm. Singh et al. included all dogs above 12 months of age and reported a median of six dogs per farm [17]. These are all higher than our result of four dogs per owner, but because TeamMate excluded any dog below 18 months of age or that were not in full work, the difference is not unexpected.

A majority of dogs were not fully trained when acquired, being either purchased as young puppies or bred by the current owner. Most dogs came at little or no cost, although some, usually fully trained adults, were occasionally bought for several thousand NZ dollars. Some farmers may want to teach dogs to work according to their own preferences, causing them to prefer self-bred and/or untrained pups over adults trained by others. However, there is no guarantee that a young pup will develop into a useful working dog and this may not be apparent until a substantial amount of time has been spent on training. In this light it makes sense that farm dogs are not seen as having much monetary value until they are older and better trained.

## Conclusions

This paper describes the TeamMate study and initial data collected from dog owners and working farm dogs at their enrolment to the study. We document previously unrecorded information about New Zealand working farm dogs and their owners and expand on previous knowledge about the dog population as well as common husbandry practices and health. Our results largely agree with previous studies while adding details that were not previously known. Further studies will involve tracking incidence rates of illness and injury, and analysis of factors that may be associated with increased risk. We are also interested to know which risk factors are associated with retirement or death of working farm dogs. This knowledge may enable us to develop guidelines for the care and husbandry of working farm dogs, helping dog owners to maintain their working dogs into later life without sacrificing the dogs’ welfare or performance.

## Methods

### Study design

TeamMate was a longitudinal study, aiming to capture data on risk factors that might affect health outcomes and career duration in working farm dogs. To capture data a veterinarian and a technician visited participating dog owners on the farm where they worked. The farm visits were carried out during distinct periods of calendar time which are referred to as ‘data collection rounds’. Six data collection rounds were carried out at roughly six monthly intervals over a period spanning May 2014 to the second half of 2018 (Fig. 1). Because of logistical issues, a small amount of overlap in dates occurred between the third and fourth data collection rounds. At each data collection round, all owners that were enrolled in the study at that point were visited. Owners and dogs were enrolled during the first three data collection rounds, with the last enrolments occurring in May 2016. As some owners moved to new farms during the course of the study, a small number of new farms were enrolled after the third data collection round. After the first and second data collection rounds, adjustments were made to the questionnaires that were used to collect data (see Additional file 1 and Additional file 2: Table S1).

At each farm visit, clinical examinations of all enrolled farm dogs were carried out by a veterinarian, and questionnaires were filled out with the help of a scribe. All veterinarians and scribes were trained to ensure data collection was performed in a standardised way, with veterinarians asked to record specific clinical signs rather than make general diagnoses. Training included a run-through of all questionnaires and how they should be completed as well as practical sessions that involved filling in the questionnaires and examining, scoring and measuring farm dogs.

### Recruitment

A convenience sample of working farm dog owners was drawn from existing clients of Vetlife, a chain of veterinary clinics on the South Island. Recruitment started in early 2014. The TeamMate study was advertised through clinic newsletters, media coverage, stalls at agricultural shows and personal invitations to those perceived to be interested. Participating dog owners became part of the ‘TeamMate Club’ and received a 5 % discount on premium dog food recommended for working dogs and 10% discounts on certain antiparasitic treatments for dogs, working dog collars, dog coats and bedding. Study results were not shared with owners until the completion of data collection. Owners were free to withdraw at any time, and otherwise remained until the end of the study.

The first time data were collected from an owner, all working farm dogs belonging to that owner that were older than 18 months old and in work at the time of data collection were enrolled in the study. Any new dogs belonging to the owner that met the eligibility criteria were enrolled each time data were collected, up to and including the third data collection round. In other words, dogs that were older than 18 months and had either been acquired between data collection rounds or had reached the age of eligibility since the previous data collection round, were enrolled. Dogs remained until the end of the study or until they died, were euthanized, retired, sold or given away, or the owner withdrew from the study.

### Data collection

#### At enrolment

##### Farms and owners

When an owner was first enrolled in the study, they were asked to provide information about themselves, the property they worked on and their working dogs. The exact questions asked about the farm and owner varied slightly between the first, second and third data collection rounds (See Additional files 1 and 2). Irrespective of data collection round, at enrolment data were collected about the property size, types of terrain present on the property and details of the types and number of stock farmed. On enrolment of owners the following data were collected: age, gender, job title, years of experience working with farm dogs, whether they bred working dogs and if so, what types of dogs and how many litters they had bred in the previous 6 months, and which types of food they had fed their working dogs during the previous 6 months.

##### Dogs

Owners were asked to provide information about each dog, skeletal size measurements were recorded, and a full physical examination was conducted. As for the farm and owner survey, the questions asked at enrolment varied somewhat between each of the three data collection rounds in which enrolments were carried out (Additional file 1 and Additional file 2: Table S1). Irrespective of data collection round, at enrolment data were captured on the age, sex, neuter status, and type of dog as well as vaccination history, skeletal size measurements and, for females, breeding history. Skeletal size measurements consisted of head length, head circumference, front leg length, hind leg length, body length and thoracic girth in centimetres. Detailed definitions of skeletal measures were included on all enrolment questionnaires (see Additional file 1).

When a dog was enrolled, dog owners were asked to provide information about any health conditions that had affected the dog’s work in the past, and clinical examinations were carried out by veterinarians. Dogs were weighed and their body condition was scored using a nine-point scale where 1 is underweight, 9 is obese, and 4 to 5 is considered ideal [21]. The physical examination included visual inspection of coat, skin, eyes, ears, teeth, footpads and nails; manual palpation of legs, tail, muscles, joints, mammary glands, testes, lymph nodes and abdomen; cardiovascular and respiratory examination with a stethoscope; and trot up to check for lameness. Range of motion was assessed in all major limb joints and the spine.

The ways in which dogs had been trained to work with stock was recorded (see Table 3 for descriptions of roles), along with the type of terrain they worked on and the types and species of stock the dog worked with (sheep, beef cattle, dairy cattle, deer, and other). Owners were asked how they acquired each dog. If the dog was not bred by the current owner, its age and level of training on arrival and any cost in money or trade was recorded. The composition of each dog’s most recent meal was recorded. If the dog was currently being given any dietary supplements or medication, the type of supplement or medication was recorded.

During the first data collection round, data were collected regarding the construction of dogs’ housing, frequency of feeding, whether each dog had the opportunity to scavenge, the types of food and water bowls used, the source of water, the modes of transport used for working dogs, where antiparasitic drugs were purchased, and whether the dog owner or the owner’s employer paid for the feeding and veterinary treatments of working dogs. After the first data collection round, most of these questions were dropped, and questions related to housing were simplified and re-worded (see Additional file 1 and Additional file 2: Table S1).

#### At follow-up

##### Farms and owners

At follow-up, dog owners were again asked to provide information about the size and terrain of the farm, how many and which types of stock were present and what types of food they had fed their working dogs since the previous data collection round (see Additional file 1 and Additional file 2: Table S1). Data collection was not repeated for information such as the dog owner’s age, gender, job title or experience.

##### Dogs

At follow-up, most of the data that were collected at enrolment were collected again (see Additional file 1 and Additional file 2: Table S1). If a dog had died, been retired or left the property between data collection rounds this was recorded and when possible the reason was noted. From the third round of data collection onwards, follow-up data were also recorded about neuter status, insurance, and council registration of all enrolled working dogs, as these may have changed between farm visits. Information collected at follow-up visits did not include data on vaccination status, working roles, the terrain a dog worked on or skeletal size measurements.

### Classification of type of dog

Dogs enrolled in TeamMate were classified based on the description given by the owner. The three most common groups were Heading dogs, Huntaways and Handy dogs. Dogs described by the owner as Beardies were classed as Huntaways, as ‘Beardie’ is a common term used to describe rough coated Huntaways. Dogs described as collies or Border Collies by the owner were classed as Heading dogs. Dogs classed as Handy dogs are dogs that can do the jobs of both Heading dogs and Huntaways, and were either described as such by their owner or were described as mixed dogs with one or both of the two main dog types in their parentage. A very small number of other types of dogs, mainly Kelpies, were listed by their reported breed.

### Coding of clinical abnormalities

Abnormalities noted on clinical examination were systematically categorised using alphanumeric codes based on the examining veterinarian’s notes. Each code consisted of a letter signifying the body system involved and up to five numbers signifying the location, symmetry, type and cause of the abnormality (see Additional file 3). Coding was carried out by a single veterinarian (LL) and checked by another person with training in veterinary health. Codes that were unclear or incomplete were re-checked by a veterinarian (LL and/or NJC). In this paper, types of abnormalities seen in fewer than 10 dogs were generally classified as ‘Other’.

### Data analysis

The data presented here were recorded on the enrolment of properties, dogs and owners to the study. As we are presenting data for descriptive purposes, no significance testing was carried out. Data were analysed using R version 3.6.0 [43]. Figures were generated using ggplot2 version 3.1.1 [44] within R, except Fig. 1 which was constructed in Microsoft PowerPoint 2016. Data were managed using Microsoft Access 2016.

## Supplementary information

**Additional file 1:** All questionnaires used to collect data for TeamMate during the first to fifth data collection rounds.

**Additional file 2: Table S1.** Types of data gathered about farming properties, working farm dog owners and working farm dogs enrolled in TeamMate. ‘Enrolment only’ refers to data that was collected on the enrolment of farms, owners or dogs, but not on follow-up. Due to changes in questionnaire design between the first, second and subsequent rounds of farm visits these are shown separately.

**Additional file 3:** An overview of the alphanumeric codes used to classify clinical abnormalities recorded in working farm dogs enrolled in TeamMate.

## Data Availability

The datasets used and/or analysed during the current study are available from the corresponding author on reasonable request.

## Abbreviations

BCS: Body condition score
CI: Confidence interval
IQR: Interquartile range
