# Supplementary material for: TeamMate: a longitudinal study of New Zealand working farm dogs. I. Methods, population characteristics and health on enrolment
Source: BMC Vet Res. 2020 Feb 17;16:59. doi: 10.1186/s12917-020-2273-2 (PMC7027279; doi:10.1186/s12917-020-2273-2)
Supplement: Supplementary file 1 — Additional file 1: All questionnaires used to collect data for TeamMate during the first to fifth data collection rounds. [file 12917_2020_2273_MOESM1_ESM.pdf]

DOG INDUCTION QUESTIONNAIRE

DATE: ______/______/2014

OWNER/HANDLER NAME:	**DOG'S NAME**:
PROPERTY NAME:	
MICROCHIP:	**STUDY ID NUMBER:**

BREED:	AGE TO CLOSEST YEAR:
GENDER: ☐ M ☐ F Spayed/Neutered Y / N	IF female: Number of litters __________
IF Spayed/Neutered - WHY?:	Number of mis-mating injections_______
INSURED: Y / N REGISTERED: Y / N	Has she ever had a vaginal prolapse Y / N

**WORMING**:	☐ The same as other dog ________________(specify name)			
Sheep Measles	Do you treat for sheep measles? Y / N			
How often?	☐ Only as a pup	☐ Once a month	☐ Every _____month(s)	
	☐ Sporadically	☐ Never	☐ Don't know	
	Where sourced: ☐ Vets	☐ Supermarket	☐ Rural supply store	☐ Ovis Management
**WORMING**:	☐ Only as a pup	☐ Once a month	☐ Every _____month(s)	
Broad spectrum	☐ Sporadically	☐ Never	☐ Don't know	
How often?	Products used:		☐ Don't know	
	Where sourced: ☐ Vets	☐ Supermarket	☐ Rural supply store	☐ Ovis Management

How often is this dog **vaccinated?**	☐ Only as a pup	☐ Sporadically	☐ Once a year	☐ Never	☐Every_____ years(s)	☐ Don't know

**TYPE OF WORK:** (tick all that apply)	☐ Head<br>☐ Catch	☐ Hunt<br>☐ Back	☐ Yard<br>☐ Dog trial
IF dog competes at dog trials:	☐ Casual (social, local trials)	☐ Competitive (to accumulate qualification points)	
Land contours worked:	☐ Flat/easy hill	☐ Steep/hill country	
TYPE OF STOCK WORKED: (tick all that apply)	☐ Sheep<br>☐ Deer	☐ Dairy cattle<br>☐ Other (please specify)	☐ Beef cattle
**WHERE ACQUIRED:**<br>☐ Self bred	☐ Other breeder		
	IF NOT self bred, age acquired:		
	Level of training when acquired:	☐ Not started ☐ Started	☐ Fully trained
	Cost if purchased:		

INDUCTION QUESTIONNAIRE CONTINUED

**FEEDING:**	☐ The same as other dog __________________(specify name)
What was this dog fed at its last meal? ☐ Meat – Specie(s)_______________ ☐ Offal – Species_________________	☐ Comm. dry brand(s)__________________________ ☐ Comm. wet brand(s)_________________________ ☐ Comm. other brand(s)________________________
How many days did this dog work stock in the last week? (All or part of a day =1 day)	
How many days was this dog fed in the last week?	
How often is this dog fed:	

In heavy work:	☐ Daily	☐ Every other day	☐ Other (please specify)
In light work	☐ Daily	☐ Every other day	☐ Other (please specify)
On days off:	☐ Daily	☐ Every other day	☐ Other (please specify)

Is this dog fed any supplements?	Y / N	Specify:
Is this dog on any medication?	Y / N	Specify:
Does this dog have the option to scavenge?	Y / N	(always tied up when not working?)
Feed bowl (made of):		Water bowl (made of):
Water source (eg: spring, bore, tank water):		

**SHELTER: (take representative photos)**	☐ The same as other dog ____________________(specify name)	
☐ Commercial or	☐ Homemade	
Run type:	☐ Cage<br>☐ Chain	☐ Other: __________________________
Run floor:	Elevated: Y / N	How high (approx)?
	☐ Wood<br>☐ Concrete	☐ Dirt<br>☐ Other:___________________
Box/Kennel:	What is floor made of?	
	What is cladding made of?	
	Elevated off the ground: Y / N	How high (approx)?
	Insulated: Y / N	Insulation material:

Does this dog have bedding in its kennel?	Y / N	Bedding material:		
Does this dog wear a coat?	Yes:	☐ at work	☐ at night	☐ No

While transported:		☐ The same as other dog ____________________(specify name)		
Truck	In cab / on deck	☐ Unrestrained	☐ Tethered	☐ Cage/kennel
Motorbike/Quad	Deck modified* Y / N	☐ Unrestrained	☐ Tethered	☐ Cage/kennel
Trailer	Deck modified* Y / N	☐ Unrestrained	☐ Tethered	☐ Cage/kennel

*modified specifically to reduce hazards which could cause injury to dogs

**COSTS** - Who pays for:		☐ The same as other dog ____________________(specify name)
Vaccination:	☐ Dog owner	☐ Other Please specify:
Worming:	☐ Dog owner	☐ Other Please specify:
Food:	☐ Dog owner	☐ Other Please specify:
Vet bills*:	☐ Dog owner	☐ Other Please specify:

*treatment for illness or injury

PHYSICAL EXAM: Indicate abnormalities on diagrams and explain next page:

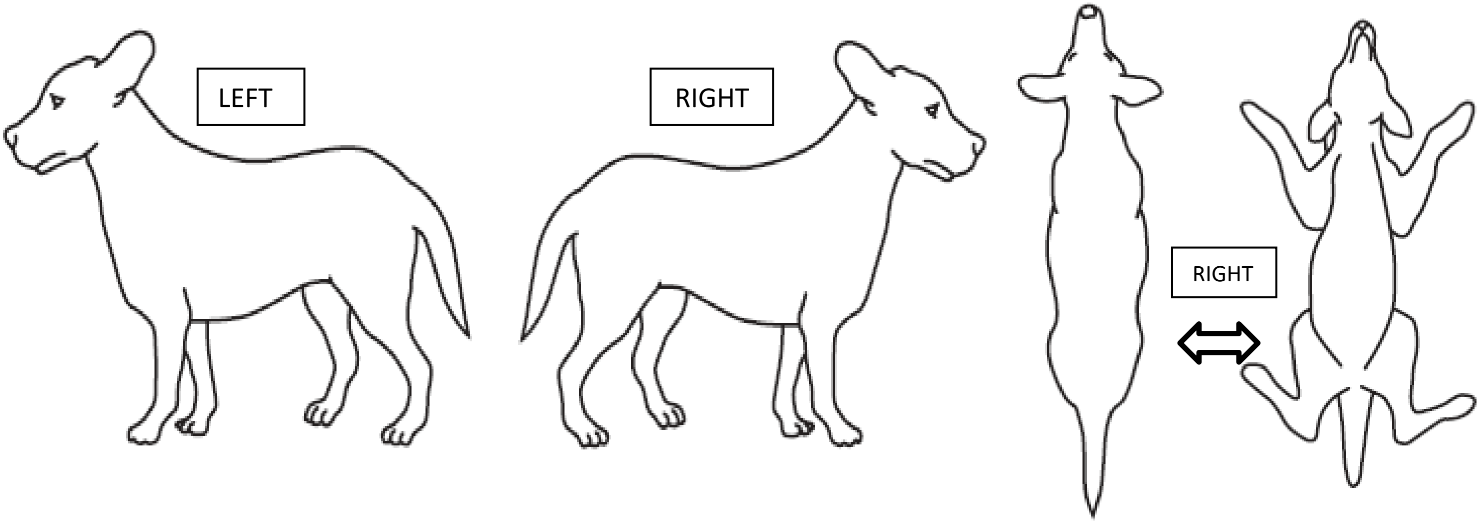

PHYSICAL EXAM	Normal	Abnormal		Normal	Abnormal
Skin/Coat	☐	☐	Respiratory	☐	☐
Pads/Nails	☐	☐	Genitourinary	☐	☐
Teeth	☐	☐	Lymph nodes	☐	☐
Ears	☐	☐	Musculoskeletal	☐	☐
Legs/Tail	☐	☐	Sound on trot up (over enough distance to assess if lame)	☐	☐
Cardiovascular	☐	☐	Joint ROM (carpus, elbow, shoulder, tarsus, stifle, hip, **tail**)	☐	☐

OCULAR EXAM – Penlight exam	Normal		Abnormal			Normal		Abnormal	
Menace reflex	L	R	L	R	Iris	L	R	L	R
Palpebral reflex	L	R	L	R	Lens	L	R	L	R
Eyelids (incl TEL)	L	R	L	R		L	R	L	R
Cornea	L	R	L	R		L	R	L	R

WT:		BCS:		Muscle condition score: (WSAVA scale)	
kg		/ 9		A B C D	
**Morphometric Measurements:**					
**1. Head length** – from level of medial canthus, equidistant between the eyes, to the external occipital protuberance.	cm	**2. Head circumference** - circumference at a point equidistant between the eyes and ears -the widest part of the head.	cm		
**3. Front leg** – From the proximal edge of the central foot pad to the point of the elbow (olecranon process) with carpus in **extension**.	cm	**4. Hind leg** – From the proximal edge of the central foot pad to the tip of the hock (dorsal tip of the calcaneal process with the tarsus in **extension.**	cm		
**5. Body length** - from the first thoracic vertebrae to the dorsal process of S1.	cm	**6. Thoracic girth** – chest circumference at the level of the xiphoid.	cm		

Record details of abnormal findings below:

Describe any previous significant injury or illness that may affect this dog's working life:

**SAMPLES TAKEN:**
☐ Photos:

		_________ / ___________ /2014
Examiner Signature	Examiner Print name	Date

DOG INDUCTION

QUESTIONNAIRE Round 2

DATE: ______/______/2015

OWNER/HANDLER NAME:	**DOG'S NAME**:
PROPERTY NAME:	

BREED:	AGE TO CLOSEST YEAR:
GENDER: ☐ M ☐ F Spayed/Neutered Y / N	IF female: Number of litters ___________
IF Spayed/Neutered - WHY?:	Number of mis-mating injections_______
	Has she ever had a vaginal prolapse Y / N

How often is this dog **vaccinated?**	☐ Only as a pup	☐ Sporadically	☐ Once a year	☐ Never	☐Every_____ years(s)	☐ Don't know

**TYPE OF WORK:** (tick all that apply)	☐ Head<br>☐ Catch	☐ Hunt<br>☐ Back	☐ Yard<br>☐ Dog trial
IF dog competes at dog trials:	☐ Casual (social, local trials)	☐ Competitive (to accumulate qualification points)	
Land contours worked:	☐ Flat/easy hill	☐ Steep/hill country	
TYPE OF STOCK WORKED: (tick all that apply)	☐ Sheep<br>☐ Deer	☐ Dairy cattle<br>☐ Other (please specify)	☐ Beef cattle
**WHERE ACQUIRED:**<br>☐ Self bred	☐ Other breeder		
	IF NOT self bred, age acquired:		
	Level of training when acquired: ☐ Not started	☐ Started	☐ Fully trained
	Cost if purchased:		

Morphometric Measurements:

**1. Head length** – from level of medial canthus, equidistant between the eyes, to the external occipital protuberance.	cm	**2. Head circumference** - circumference at a point equidistant between the eyes and ears -the widest part of the head.	cm
**3. Front leg** – From the proximal edge of the central foot pad to the point of the elbow (olecranon process) with carpus in **extension**.	cm	**4. Hind leg** – From the proximal edge of the central foot pad to the tip of the hock (dorsal tip of the calcaneal process with the tarsus in **extension.**	cm
**5. Body length** - from the first thoracic vertebrae to the dorsal process of S1.	cm	**6. Thoracic girth** – chest circumference at the level of the xiphoid.	cm

***Please attach to Round 2 questionnaire for this dog.**

WORKING DOG QUESTIONNAIRE No.2

DATE: ______/______/2015

OWNER/HANDLER NAME:	**DOG'S NAME**:
PROPERTY NAME:	

Do you have any unregistered dogs at the moment?			
Is THIS dog insured? Y / N	If yes, is it insured as:	☐ An individual OR	☐ Under general farm policy
To what value?			
Male dogs:	Has he been used for mating in the last 6 months?		
	Did this mating result in a pregnancy?		
Female dogs:	Does she come into season regularly? Y / N	How often?	
	Has she been in season in the last 6 months?		

**PARASITES**:	☐ The same as other dog ________________(specify name)	
When was the last time you treated for sheep measles?		Which product?
When was the last time you treated for intestinal worms?		Which product?
Have you noticed fleas on your dogs in the last 6 months?		
Have you treated your dogs/kennels for fleas since the last exam?		
If so, which products:		

**WORKLOAD:** Has this dogs workload increased or decreased since the last TM exam?		
Has this dogs work INTENSITY increased or decreased since the last TM exam?		
How many days did this dog work stock in the last week? (All or part of a day = 1 day)		
What type of stock has this dog worked in the last 6 months? (tick all that apply) ☐ Sheep ☐ Deer	☐ Dairy cattle ☐ Other (please specify) ____________________	☐ Beef cattle
**WT:** **kg**	**BCS:** **/ 9**	**Muscle Condition score** **A** **B** **C** **D**
**Coat Condition Score** **1** **2** **3**	**Coat Comments:**	

**FEEDING:**		☐ The same as other dog ________________(specify name)
What was this dog fed at its last meal?	☐ Meat – Specie(s)______________ ☐ Offal – Species________________	☐ Comm. dry brand(s)_________________________ ☐ Comm. wet brand(s)_________________________ ☐ Comm. other brand(s)_______________________
Is this dog fed any supplements?	Y / N Specify:	
Is this dog on any medication?	Y / N Specify:	

**SHELTER:** Has this dogs kennel/shelter arrangements changed in the last 6 months? Y / N	
Describe if necessary:	Coat? Y / N
Does this dog have bedding in its kennel at this time? Y / N	Bedding material:

PHYSICAL EXAM: Indicate abnormalities on diagrams and explain below:

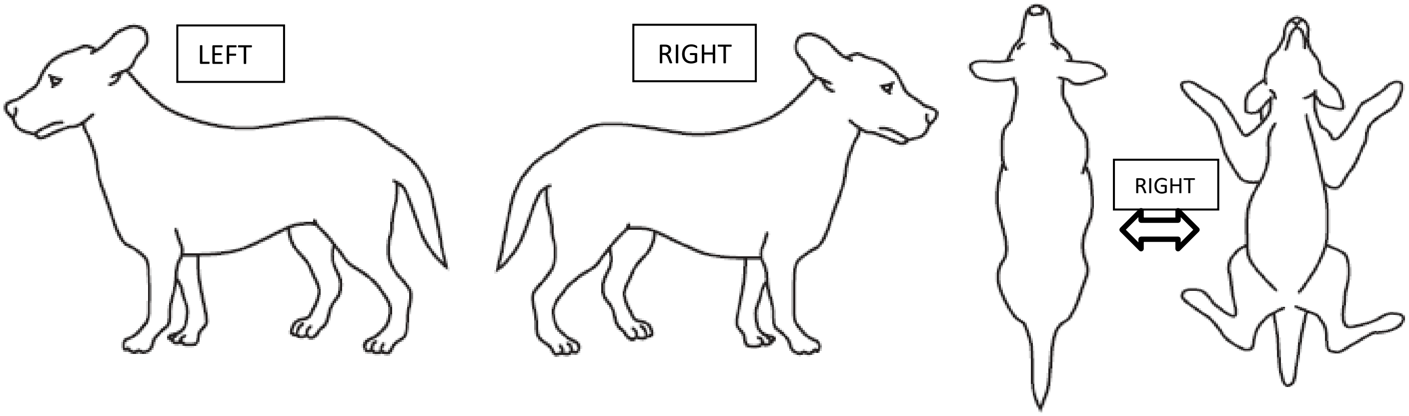

PHYSICAL EXAM	Normal	Abnormal		Normal	Abnormal
Skin/Coat	☐	☐	Respiratory	☐	☐
Pads/Nails	☐	☐	Genitourinary	☐	☐
Teeth	☐	☐	Lymph nodes	☐	☐
Ears	☐	☐	Musculoskeletal	☐	☐
Eyes	☐	☐	Sound on trot up (over enough distance to assess if lame)	☐	☐
Cardiovascular	☐	☐	Joint ROM (carpus, elbow, shoulder, tarsus, stifle, hip, **tail**)	☐	☐

Record details of abnormal findings below:

Has this dog had any injury(ies) or illness(es) since the last TM exam?

Have any of these caused this dog to miss days it would normally work?

Detail:

Examiner Signature	Examiner Print name	Date
		_________ / ___________ /2015

NEW DOG INDUCTION

QUESTIONNAIRE Round 3

DATE: ______/______/2015

OWNER/HANDLER NAME:	**DOG'S NAME**:
PROPERTY NAME:	

BREED:	COLOUR:	AGE TO CLOSEST YEAR:

GENDER: ☐ M ☐ F Spayed/Neutered Y / N	IF female: Number of litters __________
IF Spayed/Neutered - WHY?:	Number of mis-mating injections_______
	Has she ever had a vaginal prolapse Y / N

How often is this dog **vaccinated?**	☐ Only as a pup	☐ Sporadically	☐ Once a year	☐ Never	☐Every_____ years(s)	☐ Don't know

**TYPE OF WORK:** (tick all that apply)	☐ Head<br>☐ Catch	☐ Hunt<br>☐ Back	☐ Yard<br>☐ Dog trial
If dog competes at dog trials:	☐ Casual (social, local trials)	☐ Competitive (to accumulate qualification points)	
Land contours worked:	☐ Flat/easy hill	☐ Steep/hill country	
TYPE OF STOCK WORKED: (tick all that apply)	☐ Sheep<br>☐ Deer	☐ Dairy cattle<br>☐ Other (please specify)	☐ Beef cattle

**WHERE ACQUIRED:** ☐ Self bred	☐ Other breeder			
	IF NOT self bred, age acquired:			
	Level of training when acquired:	☐ Not started	☐ Started	☐ Fully trained
	Cost if purchased/traded for:			

**Morphometric Measurements:**			
**1. Head length** – from level of medial canthus, equidistant between the eyes, to the external occipital protuberance.	cm	**2. Head circumference** - circumference at a point equidistant between the eyes and ears -the widest part of the head.	cm
**3. Front leg** – From the proximal edge of the central foot pad to the point of the elbow (olecranon process) with carpus in **extension**.	cm	**4. Hind leg** – From the proximal edge of the central foot pad to the tip of the hock (dorsal tip of the calcaneal process with the tarsus in **extension.**	cm
**5. Body length** - from the first thoracic vertebrae to the dorsal process of S1.	cm	**6. Thoracic girth** – chest circumference at the level of the xiphoid.	cm

***Please attach to Round 2 questionnaire for this dog.**

TeamMate
The New Zealand working dog project

WORKING DOG EXAM.3

DATE: ______/______/2015

OWNER/HANDLER NAME:	**DOG'S NAME**:
PROPERTY NAME & ADDRESS:	
	**ID no.**

**BREEDING:**			
Male dogs:	Has he been used for mating in the last 6 months?		
	Did this mating result in a pregnancy?		
Female dogs:	Does she come into season regularly?	Y / N	How often?
	Has she been in season in the last 6 months?		What month approx?

**PARASITES**:	☐ The same as other dog ________________(specify name)
When was the last time you treated for sheep measles?	Which product?
When was the last time you treated for intestinal worms?	Which product?
Have you noticed fleas on your dogs in the last 6 months?	
Have you treated your dogs/kennels for fleas since the last exam?	
If so, which products:	

**WORKLOAD:**			
Has this dogs workload increased or decreased since the last TM exam?			
Has this dogs work INTENSITY increased or decreased since the last TM exam?			
How many days did this dog work stock in the last week? (All or part of a day = 1 day)			
What type of stock has this dog worked in the last 6 months?			
(tick all that apply)	☐ Sheep	☐ Dairy cattle	☐ Beef cattle
	☐ Deer	☐ Other (please specify) ___________________	

**FEEDING:**	☐ The same as other dog ________________(specify name)	
What was this dog fed at its last meal?	☐ Meat – Specie(s)______________	☐ Comm. dry brand(s)________________________
	☐ Offal – Specie(s)______________	☐ Comm. wet brand(s)_______________________
		☐ Comm. other brand(s)_____________________

Is this dog fed any supplements?	Y / N	Specify:
Is this dog on any medication?	Y / N	Specify:

**SHELTER:** Has this dogs kennel/shelter arrangements changed in the last 6 months? Y / N			
Describe if necessary:	Height from ground:	☐ Motel/run	☐ Kennel/chain
Does this dog have bedding in its kennel at this time? Y / N	Bedding material:		
Does this dog wear a coat? Y / N			

**WT:** **kg**	**BCS:** **/ 9**	**Muscle Condition score** A B C D
**Coat Condition Score** 1 2 3	**Coat Comments:**	

PHYSICAL EXAM: Indicate abnormalities on diagrams and explain below:

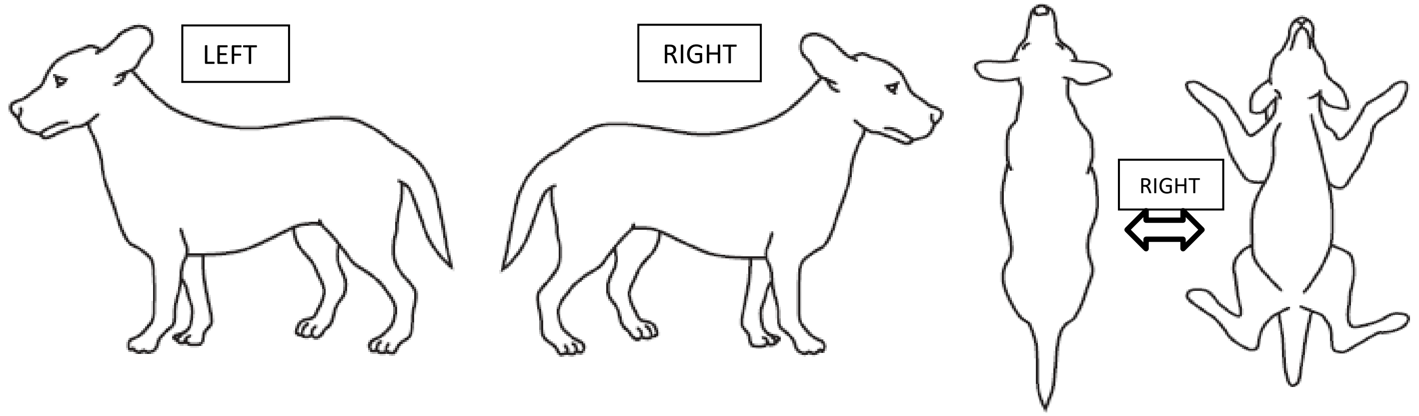

PHYSICAL EXAM	Normal	Abnormal		Normal	Abnormal
Skin/Coat	☐	☐	Respiratory	☐	☐
Pads/Nails	☐	☐	Genitourinary	☐	☐
Teeth	☐	☐	Lymph nodes	☐	☐
Ears	☐	☐	Musculoskeletal	☐	☐
Eyes	☐	☐	Sound on trot up (over enough distance to assess if lame)	☐	☐
Cardiovascular	☐	☐	Joint ROM (carpus, elbow, shoulder, tarsus, stifle, hip, tail)	☐	☐

Record details of abnormal findings below:

Has this dog had any injury(ies) or illness(es) since the last TM exam?

Have any of these caused this dog to miss days it would normally work?

Detail:

Examiner Signature	Examiner Print name	Date
		________ / __________ /2015

WORKING DOG EXAM. No.4

DATE: ______/______/2016

OWNER/HANDLER NAME:	**DOG'S NAME**:
PROPERTY NAME & ADDRESS:	
	**ID no.**

**BREEDING:**		
Male dogs:	Has he been used for mating in the last 6 months?	
	Did this mating result in a pregnancy?	
Female dogs:	Does she come into season regularly? Y / N	How often?
	Has she been in season in the last 6 months?	What month approx?

**PARASITES**:	☐ The same as other dog _________________(specify name)
When was the last time you treated for sheep measles?	Which product?
When was the last time you treated for intestinal worms?	Which product?
Have you noticed fleas on your dogs in the last 6 months?	
Have you treated your dogs/kennels for fleas since the last exam?	
If so, which products:	

**WORKLOAD:**			
Has this dogs workload increased or decreased since the last TM exam?			
Has this dogs work INTENSITY increased or decreased since the last TM exam?			
How many days did this dog work stock in the last week? (All or part of a day = 1 day)			
What type of stock has this dog worked in the last 6 months?			
(tick all that apply)	☐ Sheep	☐ Dairy cattle	☐ Beef cattle
	☐ Deer	☐ Other (please specify) ____________________	

**FEEDING:**	☐ The same as other dog _________________(specify name)	
What was this dog fed at its last meal?	☐ Meat – Specie(s)______________	☐ Comm. dry brand(s)________________________
	☐ Offal – Specie(s)______________	☐ Comm. wet brand(s)_______________________
		☐ Comm. other brand(s)______________________

Is this dog fed any supplements?	Y / N	Specify:
Is this dog on any medication?	Y / N	Specify:

**SHELTER:** Has this dogs kennel/shelter arrangements changed in the last 6 months? Y / N			
Describe if necessary:	Height from ground:	☐ Motel/run	☐ Kennel/chain
Does this dog have bedding in its kennel at this time? Y / N	Bedding material:		
Does this dog wear a coat? Y / N			

**WT:** kg	**BCS:** / 9	**Muscle Condition score:** A B C D
**Coat Condition Score** 1 2 3	**Coat Comments:**	

PHYSICAL EXAM: Indicate abnormalities on diagrams and explain below:

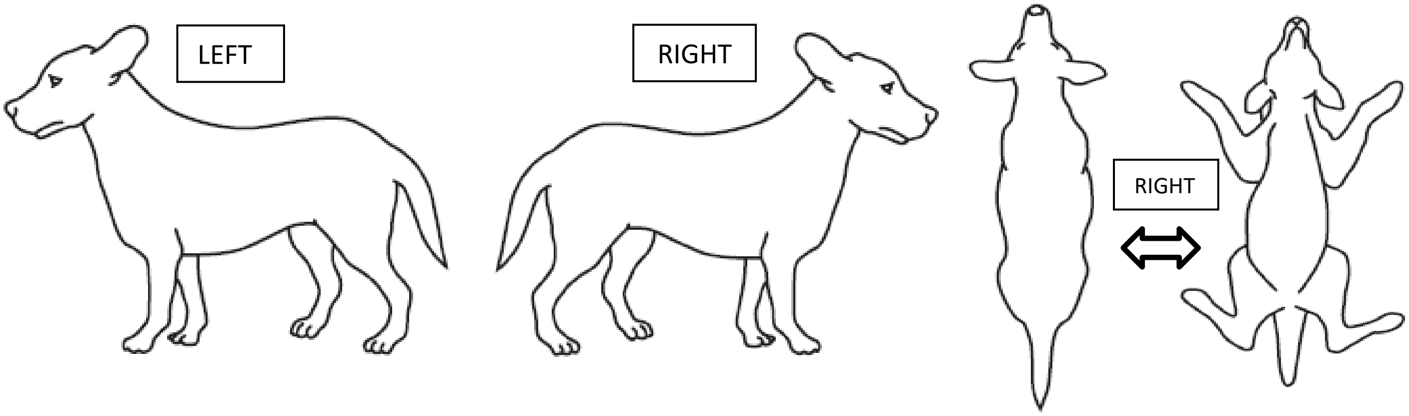

PHYSICAL EXAM:	Normal	Abnormal		Normal	Abnormal
Skin/Coat	☐	☐	Respiratory	☐	☐
Pads/Nails	☐	☐	Genitourinary	☐	☐
Teeth	☐	☐	Lymph nodes	☐	☐
Ears	☐	☐	Musculoskeletal	☐	☐
Eyes	☐	☐	Sound on trot up (over enough distance to assess if lame)	☐	☐
Cardiovascular	☐	☐	Joint ROM (carpus, elbow, shoulder, tarsus, stifle, hip, tail)	☐	☐

Record details of abnormal findings below:

Has this dog had any injury(ies) or illness(es) since the last TM exam?

Have any of these caused this dog to miss days it would normally work?

Detail:

		_________ / ___________ /2016
Examiner Signature	Examiner Print name	Date

TeamMate
The New Zealand working dog project

WORKING DOG EXAM. No.5

DATE: ______/______/2017

OWNER/HANDLER NAME:	**DOG'S NAME:**
PROPERTY NAME & ADDRESS:	
	**Heyrex monitor:**

**BREEDING:**		
Male dogs:	Has he been used for mating in the last 6 months?	
	Did this mating result in a pregnancy?	
Female dogs:	Does she come into season regularly? Y / N	How often?
	Has she been in season in the last 6 months?	What month approx?

**PARASITES:**	☐ The same as other dog __________________(specify name)
When was the last time you treated for sheep measles?	Which product?
When was the last time you treated for intestinal worms?	Which product?
Have you noticed fleas on your dogs in the last 6 months?	
Have you treated your dogs/kennels for fleas since the last exam?	
If so, which products:	

**WORKLOAD:**			
Has this dogs workload increased or decreased since the last TM exam?			
Has this dogs work INTENSITY increased or decreased since the last TM exam?			
How many days did this dog work stock in the last week? (All or part of a day = 1 day)			
What type of stock has this dog worked in the last 6 months?			
(tick all that apply)	☐ Sheep	☐ Dairy cattle	☐ Beef cattle
	☐ Deer	☐ Other (please specify) _____________________	

**FEEDING:**	☐ The same as other dog __________________(specify name)	
What was this dog fed at its last meal?	☐ Meat – Specie(s)_______________	☐ Comm. dry brand(s)_________________________
	☐ Offal – Specie(s)_______________	☐ Comm. wet brand(s)________________________
		☐ Comm. other brand(s)_______________________

Is this dog fed any supplements?	Y / N	Specify:
Is this dog on any medication?	Y / N	Specify:

**SHELTER:** Has this dogs kennel/shelter arrangements changed in the last 6 months? Y / N			
Describe if necessary:	Height from ground:	☐ Motel/run	☐ Kennel/chain
Does this dog have bedding in its kennel at this time? Y / N	Bedding material:		
Does this dog wear a coat? Y / N			

**WT:** kg	**BCS:** / 9	**Muscle Condition score:** A B C D
**Coat Condition Score** 1 2 3	**Coat Comments:**	

PHYSICAL EXAM: Indicate abnormalities on diagrams and explain below:

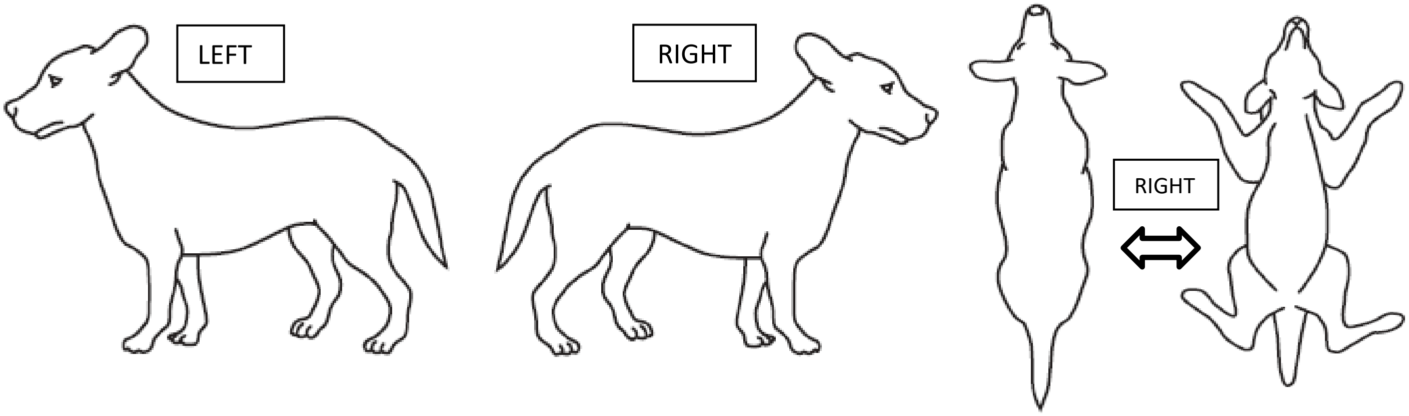

PHYSICAL EXAM:	Normal	Abnormal		Normal	Abnormal
Skin/Coat	☐	☐	Respiratory	☐	☐
Pads/Nails	☐	☐	Genitourinary	☐	☐
Teeth	☐	☐	Lymph nodes	☐	☐
Ears	☐	☐	Musculoskeletal	☐	☐
Eyes	☐	☐	Sound on trot up (over enough distance to assess if lame)	☐	☐
Cardiovascular	☐	☐	Joint ROM (carpus, elbow, shoulder, tarsus, stifle, hip, tail)	☐	☐

Record details of abnormal findings below:

Has this dog had any injury(ies) or illness(es) since the last TM exam?

Have any of these caused this dog to miss days it would normally work?

Detail:

		_________ / ___________ /2017
Examiner Signature	Examiner Print name	Date

OWNER/HANDLER INFORMATION

DATE: ______/______/_______

OWNER/HANDLER NAME:	ID no.
PROPERTY NAME:	
ADDRESS:	
Email Address:	

**OWNER/HANDLER:**			
Age to closest year:	☐ <20 years	☐ 41-50 years	☐ > 70 years
	☐ 21 - 30 years	☐ 51-60 years	
	☐ 31 - 40 years	☐ 61-70 years	
Gender:	☐ Male	☐ Female	
Job title:<br>(Tick all that apply)	☐ Owner	☐ Manager	☐ Employee
How many years experience do you have working dogs?			
Have you attended any formal dog training courses?		☐ Yes	☐ No
Do you breed working dogs?		☐ Yes	☐ No
How many litters of pups have you bred in the last year?			
Which breed(s)?			
**PROPERTY:**	Property size in hectares:		
	Contour of <u>farmed</u> land:<br>*Tick all that apply	☐ Flat/Easy hill	☐ Steep/Hill country
**Stock numbers:**	Sheep:		
	Cattle Beef:	Dairy dry stock:	Dairy milking:
	Deer:		
	Other (please specify):		

**Types of foods fed over the last 6 months:**			
Meat? Y / N	Specie(s):	☐ Home sourced	☐ Purchased
		☐ Fresh	☐ Frozen
Offal? Y / N	Specie(s):	☐ Fresh	☐ Cooked
Commercial dog	☐ Commercial dry	Brand(s):	
Food? Y / N	☐ Commercial wet	Brand(s):	
	☐ Commercial other	Brand(s):	

List of all dogs over the age of **<u>2 months</u>** owned by you at the property at each visit (not just the dogs in the study):

	Name	Breed	Age	Colour
1				
2				
3				
4				
5				
6				
7				
8				
9				
10				
11				
12				
13				
14				
15				
16				
17				
18				
19				
20				

Litters of un-weaned pups:

	Bitch's name	Breed of pups	Number of pups in litter	Approx age
1				
2				
3				
4				
5				

OWNER/HANDLER EXAM 2

DATE: ______/______/_2015

**OWNER/HANDLER NAME:**	ID no.

Have you or are you planning to mate any bitches this season?			
Have you had any litters of pups this year?			
Which breed(s)?			
**PROPERTY:**	Property size in hectares:		
☐ UNCHANGED	Contour of farmed land: *Tick all that apply	☐ Flat/Easy hill	☐ Steep/Hill country
**Stock numbers:**	Sheep:		
	Cattle Beef:	Dairy dry stock:	Dairy milking:
☐ **UNCHANGED**	Deer:		
	Other (please specify)		

**Types of foods fed over the last 6 months:**			
Meat? Y / N	Specie(s):	☐ Home sourced	☐ Purchased
		☐ Fresh	☐ Frozen
Offal? Y / N	Specie(s):	☐ Fresh	☐ Cooked
Commercial dog	☐ Commercial dry	Brand(s):	
Food? Y / N	☐ Commercial wet	Brand(s):	
	☐ Commercial other	Brand(s):	

List of all dogs over the age of 2 months owned by you at the property at each visit (not just the dogs in the study):

	Name	Breed	Age	Colour
1				
2				
3				
4				
5				
6				
7				
8				
9				
10				
**Litters of unweaned pups:**				
	**Bitch's name**	**Breed of pups**	**Number of pups in litter**	**Approx age**
1				
2				

Continued from previous page if necessary:

	Name	Breed	Age	Colour
1				
2				
3				
4				
5				
6				
7				
8				
9				
10				

Litters of unweaned pups:

	Bitch's name	Breed of pups	Number of pups in litter	Approx age
1				
2				

NEW OWNER/HANDLER/PROPERTY
INDUCTION QUESTIONNAIRE ROUND 3

DATE: ______/______/_______

OWNER/HANDLER NAME:	ID no.
PROPERTY NAME:	
ADDRESS:	
Email address:	

**OWNER/HANDLER:**			
Age to closest year:	☐ <20 years	☐ 41-50 years	☐ > 70 years
	☐ 21 - 30 years	☐ 51-60 years	
	☐ 31 - 40 years	☐ 61-70 years	
Gender:	☐ Male	☐ Female	
Job title:<br>(Tick all that apply)	☐ Owner	☐ Manager	☐ Employee
How many years experience do you have working dogs?			
Have you attended any formal training courses?		☐ Yes	☐ No
Do you breed working dogs?		☐ Yes	☐ No
How many litters of pups have you bred in the last year?			
Which breed(s)?			

**PROPERTY:**	Property size in hectares:		
	Contour of farmed land:<br>*Tick all that apply	☐ Flat/Easy hill	☐ Steep/Hill country
Stock numbers:	Sheep:		
	Cattle Beef:	Dairy dry stock:	Dairy milking:
	Deer:		
	Other (please specify)		

**Types of foods fed over the last 6 months:**				
Meat?	Y / N	Specie(s):	☐ Home sourced	☐ Purchased
			☐ Fresh	☐ Frozen
Offal?	Y / N	Specie(s):	☐ Fresh	☐ Cooked
		☐ Commercial dry	Brand(s):	
		☐ Commercial wet	Brand(s):	
		☐ Commercial other	Brand(s):	

TeamMate
The New Zealand working dog project

OWNER/HANDLER/PROPERTY EXAM.3

DATE: _____/_____/2015

**OWNER/HANDLER NAME:**	ID no.
**PROPERTY NAME AND ADDRESS:**	

**PROPERTY:**	Property size in hectares:		
☐ UNCHANGED	Contour of farmed land:		
	*tick all that apply	☐ flat/easy hill	☐ steep/hill country
**STOCK NUMBERS:**	Sheep:		
☐ UNCHANGED	Cattle beef:	Dairy drystock:	Dairy milking:
	Deer:		
	Other (please specify):		

**TYPES OF FOODS FED OVER THE LAST SIX MONTHS:**				
Meat?	Y / N	Specie(s):	☐ Home sourced	☐ Purchased
			☐ Fresh	☐ Frozen
Offal?	Y / N	Specie(s):	☐ Fresh	☐ Cooked
Commercial dog food?	Y / N	☐ Commercial dry	Brand(s):	
		☐ Commercial wet	Brand(s):	
		☐ Commercial other	Brand(s):	

Notes:

List all dogs over the age of 2 months owned by you at the property at each visit (not just the dogs in the study):

	Name	Breed HW/HD/BE/ HY/KP	Gender M/F	Neutered Y/N	If dog is neutered – why?	Age	Colour	Insured Y/N	Value of insurance	Registered Y/N	Deceased/sold/ given away OR OTHER	If deceased/sold/ given away – how or why? Or if present but not examined – why?
1												
2												
3												
4												
5												
6												
7												
8												
9												
10												
11												
12												
13												
14												

BREED: HW – Huntaway : HD – heading dog : BE – Beardie : HY – Handy : KP – Kelpie

Any litters of un-weaned pups:

	Bitch's name	Breed of pups	Number of pups in litter	Approx. age
1				
2				
3				

TeamMate
The New Zealand working dog project

OWNER/HANDLER/PROPERTY EXAM.4

DATE: _____/_____/2016

**OWNER/HANDLER NAME:**	ID no.
**PROPERTY NAME AND ADDRESS:**	

**PROPERTY:** ☐ UNCHANGED	Property size in hectares:		
	Contour of farmed land:		
	*tick all that apply	☐ flat/easy hill	☐ steep/hill country
**STOCK NUMBERS:** ☐ UNCHANGED	Sheep:		
	Beef cattle:	Dairy drystock:	Dairy milking:
	Deer:		
	Other (please specify):		

**TYPES OF FOODS FED OVER THE LAST SIX MONTHS:**				
Meat?	Y / N	Specie(s):	☐ Home sourced	☐ Purchased
			☐ Fresh	☐ Frozen
Offal?	Y / N	Specie(s):	☐ Fresh	☐ Cooked
Commercial dog food?	Y / N	☐ Commercial dry	Brand(s):	
		☐ Commercial wet	Brand(s):	
		☐ Commercial other	Brand(s):	

Notes:

List **ALL DOGS** over the age of 2 months owned by you at the property at each visit (not just the dogs in the study):

	**Name**	**Breed** HW/HD/BE /HY/KP	**Gender M/F**	**Neutered Y/N**	**If dog is neutered – why?**	**Age**	**Colour**	**Insured Y/N**	**Value of insurance**	**Registered Y/N**	**Deceased/sold/ given away OR OTHER**	**If deceased/sold/ given away – how or why? Or if present but not examined – why?**
**1**												
**2**												
**3**												
**4**												
**5**												
**6**												
**7**												
**8**												
**9**												
**10**												
**11**												
**12**												
**13**												
**14**												

BREED: HW – Huntaway : HD – heading dog : BE – Beardie : HY – Handy : KP – Kelpie

Any litters of un-weaned pups:

	**Bitch's name**	**Breed of pups**	**Number of pups in litter**	**Approx. age**
**1**				
**2**				

TeamMate
The New Zealand working dog project

OWNER/HANDLER/PROPERTY EXAM.5

DATE: _____/_____/2017

**OWNER/HANDLER NAME:**	ID no.
**PROPERTY NAME AND ADDRESS:**	
**EMAIL ADDRESS:**<br>*(So we can send TeamMate farmers information regarding their dogs)*	

**PROPERTY:**	Property size in hectares:		
☐ UNCHANGED	Contour of farmed land:		
	*tick all that apply	☐ flat/easy hill	☐ steep/hill country
**STOCK NUMBERS:**	Sheep:		
☐ UNCHANGED	Beef cattle:	Dairy drystock:	Dairy milking:
	Deer:		
	Other (please specify):		

**TYPES OF FOODS FED OVER THE LAST SIX MONTHS:**				
Meat?	Y / N	Specie(s):	☐ Home sourced	☐ Purchased
			☐ Fresh	☐ Frozen
Offal?	Y / N	Specie(s):	☐ Fresh	☐ Cooked
Commercial dog food?	Y / N	☐ Commercial dry	Brand(s):	
		☐ Commercial wet	Brand(s):	
		☐ Commercial other	Brand(s):	

Notes:

List **ALL DOGS** over the age of 2 months owned by you at the property at each visit (not just the dogs in the study):

	Name	Breed HW/HD/BE /HY/KP	Gende r M/F	Neute red Y/N	If dog is neutered – why?	Age	Colour	Insured Y/N	Value of insurance	Register ed Y/N	Deceased/sold/ given away OR OTHER	If deceased/sold/ given away – how or why? Or if present but not examined – why?
**1**												
**2**												
**3**												
**4**												
**5**												
**6**												
**7**												
**8**												
**9**												
**10**												
**11**												
**12**												
**13**												
**14**												

BREED: HW – Huntaway : HD – heading dog : BE – Beardie : HY – Handy : KP – Kelpie

Any litters of un-weaned pups:

	Bitch's name	Breed of pups	Number of pups in litter	Approx. age
**1**				
**2**				
