# Supplementary material for: TeamMate: a longitudinal study of New Zealand working farm dogs. I. Methods, population characteristics and health on enrolment
Source: BMC Vet Res. 2020 Feb 17;16:59. doi: 10.1186/s12917-020-2273-2 (PMC7027279; doi:10.1186/s12917-020-2273-2)
Supplement: Supplementary file 2 — Additional file 2: Table S1. Types of data gathered about farming properties, working farm dog owners and working farm dogs enrolled in TeamMate. ‘Enrolment only’ refers to data that was collected on the enrolment of farms, owners or dogs, but not on follow-up. Due to changes in questionnaire design between the first, second and subsequent rounds of farm visits these are shown separately. [file 12917_2020_2273_MOESM2_ESM.docx]

Supplementary table 1: Types of data gathered about farming properties, working farm dog owners and working farm dogs enrolled in TeamMate. ‘Enrolment only’ refers to data that was collected on the enrolment of farms, owners or dogs, but not on follow-up. Due to changes in questionnaire design between the first, second and subsequent rounds of farm visits these are shown separately.

| **Level and type of information recorded** | **Time of data collection** | | | |
| --- | --- | --- | --- | --- |
|  | **Enrolment only** | **Data collection round** | | |
|  |  | **First** | **Second** | **Third and later** |
| General |  |  |  |  |
| Date of visit |  | x | x | x |
| Property |  |  |  |  |
| Property name and address | x |  |  |  |
| Property size |  | x | x | x |
| Stock types and numbers |  | x | x | x |
| Dog owner |  |  |  |  |
| Name and contact information | x |  |  |  |
| Age, gender, job description | x |  |  |  |
| Experience and training | x |  |  |  |
| Dog feeding practices |  | x | x | x |
| Dog breeding practices |  | x | x |  |
| Current un–weaned litters |  | x | x | x |
| Presence of unregistered dogs on farm |  |  | x |  |
| Dogs |  |  |  |  |
| Age, sex, signalment |  | x | x | x |
| Fate of dog (if no longer on farm) |  |  | x | x |
| Breeding history (female) | x |  |  |  |
| Vaccination history | x |  |  |  |
| Neuter status and reason for neutering |  | x | x | x |
| Council registration |  | x |  | x |
| Insurance coverage |  | x | x | x |
| Type of insurance |  |  | x |  |
| Value of insurance |  |  | x | x |
| Work type, terrain, stock types | x |  |  |  |
| Dog source, training level and cost | x |  |  |  |
| Body size measurements | x |  |  |  |
| Kennel type and elevation |  | x | x | x |
| Kennel insulation and building materials |  | x |  |  |
| Bedding and coat use |  | x | x | x |
| Dog transportation |  | x |  |  |
| Employer contributions to dog food, vaccinations and veterinary treatments |  | x |  |  |
| Parasite treatments |  | x | x | x |
| Parasite treatments source |  | x |  |  |
| Recent workload |  | x | x | x |
| Meal frequency |  | x |  |  |
| Most recent meal composition |  | x | x | x |
| Medication or supplements given |  | x | x | x |
| Body weight and condition score |  | x | x | x |
| Recent breeding performance (males) |  | x | x | x |
| Recent oestrus cycle (females) |  | x | x | x |
| Medical history |  | x | x | x |
| Clinical examination |  | x | x | x |
